# An overview of systematic reviews investigating clinical features for diagnosing neck pain and its associated disorders

**DOI:** 10.1080/10669817.2024.2436403

**Published:** 2024-12-13

**Authors:** Brandon C. Williams, Scott W. Lowe, Ryan C. McConnell, Joshua A. Subialka

**Affiliations:** a Orthopaedic Manual Physical Therapy Fellowship Program, Upstream Rehabilitation Institute, Smyrna, GA, USA; b Department of Physical Therapy, Philadelphia College of Osteopathic Medicine, Georgia, Suwanee, GA, USA; c Department of Physical Therapy, College of Pharmacy and Health Sciences, Belmont University, Nashville, TN, USA; d Department of Physical Therapy, College of Health Sciences, Midwestern University, Glendale, AZ, USA

**Keywords:** neck pain, whiplash-associated disorders, cervical instability, diagnosis, examination

## Abstract

**Background:**

Neck pain is a common condition that is often difficult to diagnose. Previous literature has investigated diagnostic accuracy of examination measures, but the strength and clinical applicability are limited. This overview of systematic reviews aimed to investigate clinical features for diagnosing neck pain and its associated disorders.

**Methods:**

An overview of systematic reviews was conducted searching four electronic databases for systematic reviews evaluating diagnostic criteria for neck pain. Quality and risk of bias were assessed using the AMSTAR 2 and ROBIS. Clinical features for neck pain were investigated for diagnostic utility.

**Results:**

Twenty-seven systematic reviews were included. Hand radiculopathy and numbness have good specificities (0.89–0.92) for facet and uncinate joint hypertrophy. For facet-related dysfunction, the extension rotation test (ERT) and manual assessment have good sensitivities and moderate-good specificities. Positive ERT combined with positive manual assessment findings (+LR = 4.71; Sp = 0.83) improves diagnostic accuracy compared to positive ERT alone (+LR = 2.01; Sp = 0.59). Canadian C-spine Rules and Nexus criteria have excellent validity in screening for cervical fracture or instability. Imaging appears to have validity in diagnosing ligamentous disruption or fractures but lacks clarity on predicting future neck pain. Increased fatty infiltrates have been found with whiplash-associated disorders and mechanical neck pain.

**Conclusions:**

This review found limited indicators providing strong diagnostic utility for diagnosing neck pain. Strength of recommendations are limited by heterogeneous outcomes, methodology, and classification systems. Future research should investigate new differential diagnostic criteria for specific structures contributing to neck pain.

## Introduction

Neck pain and its associated disorders (NAD) are common musculoskeletal disorders and leading causes of global disability, with 30–50% of adults experiencing neck pain in a given year, and 50–85% not achieving complete resolution of symptoms [1–3]. NAD may present without an identified anatomical source, making accurate diagnosis difficult [4]. Guidelines for neck pain have emphasized using a biopsychosocial framework with impairment/function-related diagnoses, rather than specific mechanical diagnoses, but conclude that evidence regarding the diagnostic utility of current tests and measures is lacking [5]. Previous research has found no definitive relationships between complaints of neck pain and imaging findings and acknowledges there is a lack of consensus on gold standards [6].

Clinical questioning, such as patient history and red flag screening, can assess the appropriateness of treatment, justify medical referral, and direct further assessment [7,8]. Red flag screening for serious pathologies, such as fracture or ligamentous instability, may indicate a need for imaging [7,9] Movement assessments may be used to assess pain location, referral sources, and mobility of structures that are associated with NAD [5]. Subjective interviewing and objective clinical measurements may inform an accurate diagnosis, optimizing outcomes and meeting patient expectations [5,10,11]. Objective clinical measurements may include assessments of range of motion (ROM), strength, coordination, postural observation, manual assessment of accessory and physiologic joint mobility, and imaging [12,13]. However, the heterogeneity and multifactorial nature of NAD, along with conflicting recommendations for diagnostic testing, pose challenges to accurately diagnosing specific structures [5,14–17].

Healthcare providers of all disciplines and experience levels may benefit from improved diagnostic capabilities and recognition of common clinical features relating to NAD [18–20]. Thus, this overview of systematic reviews aimed to investigate clinical features for diagnosing NAD and identifying when specific mechanical causes are present. NAD definitions from Haldeman et al [2]. were used and can be seen in Table 1. Additionally, we explored specific mechanical causes of neck pain for NAD patients, with definitions and examples provided in Table 2 [21–27].

**Table 1.** Neck Pain and its Associated Disorders Definitions

NAD Classifications^2^	Definition
Grade I	No signs or symptoms suggestive of major structural pathology and no or minor interference with activities of daily living; will likely respond to minimal intervention such as reassurance and pain control; does not require intensive investigations or ongoing treatment.
Grade II	No signs or symptoms of major structural pathology, but major interference with activities of daily living; requires pain relief and early activation/intervention aimed at preventing long-term disability.
Grade III	No signs or symptoms of major structural pathology, but presence of neurologic signs such as decreased deep tendon reflexes, weakness, and/or sensory deficits; might require investigation and, occasionally, more invasive treatments.
Grade IV	Signs or symptoms of major structural pathology, such as fracture, myelopathy, neoplasm, or systemic disease; requires prompt investigation and treatment.

a Table adapted from Haldeman et al. [2]

**Table 2.** Mechanical Neck Pain Definitions

Specific cause of neck pain	Description	Examples
Mechanical neck pain [21–23]	Pain associated with muscular, joint, and/or neural dysfunction	Muscle strain/tear, trigger point referral, osteoarthritis, stenosis, facet arthropathy/dysfunction, disc referral
Whiplash associated disorder (WAD) [24]	A combination of symptoms affecting the neck followed by an accident with an acceleration–deceleration mechanism	Motor vehicle accident, fall
Cervical instability [24,26,27]	The inability of the cervical spine to maintain its normal pattern of displacement between the vertebrae under physiologic loads, so that articular compromise renders joints vulnerable to disruption	Vertebral fracture, joint dislocation, upper cervical ligamentous sprain/rupture

## Methods

This review was conducted following the Preferred Reporting Items for Systematic Review and Meta-Analysis (PRISMA) guidelines [28] and registered a priori (PROSPERO registration number CRD42022325045).

### Search strategy

A literature search was conducted for systematic reviews published between January 2016 and June 2024 using CINAHL, MEDLINE, Embase, and Web of Science databases. See Supplemental Appendix A for search strategy details. Included systematic reviews were cross-referenced for other reviews that were published in the date range but missed in the search to ensure no relevant systematic reviews were excluded.

### Study selection criteria

Article selection was conducted by two independent reviewers (SWL and BCW) with a third reviewer (JAS) resolving conflicts. Systematic reviews, with or without meta-analysis, examining clinical features of NAD such as subjective reports, movement-based clinical tests, or laboratory tests, were included for review (Table 3). Due to the heterogeneity of cervical spine conditions, systematic reviews investigating neck pain and dysfunction caused by the cervical spine or surrounding structures (e.g. muscle, joint, nerve, intervertebral disc, etc.) were eligible for inclusion.

**Table 3.** Eligibility Criteria

Systematic Review Eligibility
Systematic reviews of randomized controlled trials
Assesses pathoanatomical diagnosis of neck pain, including neurological compromise or other body regions referring to the neck
Human subjects
Age ≥ 18 years old
Full text available in English

### Data management

Covidence, a web-based data management tool for systematic reviews (Veritas Health Innovation Ltd, Melbourne, Australia), was used for article screening and data extraction. Two independent reviewers (SWL and BCW) performed title and abstract screening, full-text screening, and data extraction. Disagreements were resolved by a third reviewer (JAS).

### Data extraction

Two reviewers (SWL and BCW) individually recorded diagnostic clinical features related to NAD, including diagnostic utility (Table 4) and other NAD features and risk factors (Supplemental Appendix B). Narrative summaries were extracted, as appropriate (Supplemental Appendix C).

**Table 4.** NAD Diagnostic Accuracy

Mechanical Neck Pain		
Diagnostic Test	Studies (Lead author and year)	Diagnostic Validity/Accuracy Statistics Reported
Self-Report Items with Subjective History	Mizer [32]	**Degenerative disc disease**
		*Headache*
		DOR (95% CI): 0.43 (0.36, 0.50)
		*Neck stiffness*
		DOR (95% CI): 0.62 (0.56, 0.68)
		*Shoulder referral*
		DOR (95% CI): 0.60 (0.54,0.67)
		*Hand radiculopathy*
		DOR (95% CI): 0.57 (0.47, 0.67)
		*Hand numbness*
		DOR (95% CI):0.57 (0.47, 0.68)
		**Facet joint hypertrophy**
		*Headache*
		Sp: 0.65 (95% CI: 0.59, 0.71)
		Sn: 0.28 (95% CI: 0.21, 0.37)
		+LR: 0.81 (95% CI: 0.57, 1.14)
		-LR: 1.10 (95% CI: 0.95, 1.28)
		PPV: 0.32 (95% CI: 0.24, 0.42)
		NPV: (95% CI: 0.54, 0.67)
		*Neck stiffness*
		Sp: 0.40 (95% CI: 0.34, 0.47)
		Sn: 0.71 (95% CI: 0.63, 0.79)
		+LR: 1.20 (95% CI: 1.02, 1.41)
		-LR: 0.71 (95% CI: 0.51, 0.98)
		PPV: 0.41 (95% CI: 0.34, 0.48)
		NPV: 0.71 (95% CI: 0.62, 0.78)
		*Shoulder referral*
		Sp: 0.64 (95% CI: 0.57, 0.70)
		Sn: 0.41 (95% CI: 0.32, 0.50)
		+LR: 1.13 (95% CI: 0.85, 1.50)
		-LR: 0.93 (95% CI: 0.77, 1.11)
		PPV: 0.40 (95% CI: 0.31, 0.49)
		NPV: 0.64 (95% CI: 0.57, 0.71)
		*Hand radiculopathy*
		Sp: 0.89 (95% CI: 0.84, 0.93)
		Sn: 0.14 (95% CI: 0.09, 0.21)
		+LR: 1.26 (95% CI: 0.69, 2.30)
		-LR: 0.99 (95% CI: 0.89, 1.06)
		PPV: 0.42 (95% CI: 0.28, 0.58)
		NPV: 0.64 (95% CI: 0.58, 0.69)
		*Hand numbness*
		Sp: 0.90 (95% CI: 0.85, 0.94)
		Sn: 0.10 (95% CI: 0.06, 0.17)
		+LR: 0.99 (95% CI: 0.50, 1.94)
		-LR: 1.00 (95% CI: 0.93, 1.08)
		PPV: 0.36 (95% CI: 0.22, 0.53)
		NPV: 0.63 (95% CI: 0.58, 0.69)
		**Uncinate joint hypertrophy**
		*Headache*
		Sp: 0.66 (95% CI: 0.59, 0.73)
		Sn: 0.30 (95% CI: 0.24, 0.39)
		+LR: 0.91 (95% CI: 0.66, 1.26)
		-LR: 1.05 (95% CI: 0.90, 1.22)
		PPV: 0.42 (95% CI: 0.33, 0.51)
		NPV: 0.55 (95% CI: 0.48, 0.61)
		*Neck stiffness*
		Sp: 0.41 (95% CI: 0.34, 0.48)
		Sn: 0.70 (95% CI: 0.61, 0.77)
		+LR: 1.17 (95% CI: 1.00, 1.38)
		-LR: 0.75 (95% CI: 0.55, 1.01)
		PPV: 0.48 (95% CI: 0.41, 0.55)
		NPV: 0.63 (95% CI: 0.54, 0.71)
		*Shoulder referral*
		Sp: 0.66 (95% CI: 0.59, 0.73)
		Sn: 0.44 (95% CI: 0.36, 0.52)
		+LR: 1.23 (95% CI: 0.98, 1.70)
		-LR: 0.85 (95% CI: 0.71, 1.02)
		PPV: 0.5 (95% CI: 0.42, 0.59)
		NPV: 0.6 (95% CI: 0.53, 0.66)
		*Hand radiculopathy*
		Sp: 0.91 (95% CI: 0.85, 0.94)
		Sn: 0.15 (95% CI: 0.10, 0.22)
		+LR: 1.59 (95% CI: 0.87, 2.89)
		-LR: 0.94 (95% CI: 0.86, 1.02)
		PPV: 0.55 (95% CI: 0.40, 0.70)
		NPV: 0.58 (95% CI: 0.52, 0.63)
		*Hand numbness*
		Sp: 0.92 (95% CI: 0.87, 0.95)
		Sn: 0.13 (95% CI: 0.08, 0.19)
		+LR: 1.54 (95% CI: 0.81, 2.95)
		-LR: 0.95 (95% CI: 0.88, 1.03)
		PPV: 0.55 (95% CI: 0.38, 0.70)
		NPV: 0.57 (95% CI: 0.52, 0.63)
Manual Assessment	Usunier [16]	**Pooled Validity**
		*Passive intersegmental motion testing for identifying pain*
		Sn: 0.90 (95% CI: 0.85, 0.94)
		Sp: 0.73 (95% CI: 0.65, 0.81)
		*Mechanical sensitivity for identifying joint pain*
		Sn: 0.88 (95% CI: 0.78, 0.95)
		Sp: 0.61 (95% CI: 0.50, 0.71)
		p < 0.01)
	Lemeunier [20]	***Validity***
		*Static tenderness to palpation at C2-7 paraspinal muscles compared to facet blocks*
		Sn: 0.94
		Sp: 0.73
		*Joint palpation compared to medial branch nerve blockades for facet joint pain*
		Sn: 0.89–0.92
		Sp: 0.47–0.71
Extension-Rotation Test (ERT)	Lemeunier [18]	***Validity***
		Sn: 82.7% (95% CI: 70.3, 90.6)
		Sp: 58.9% (95% CI: 47.5, 69.5)
		+LR: 2.01 (95% CI: 1.49, 2.72)
		-LR: 0.29 (95% CI: 0.16, 0.55)
Extension-Rotation Test Combined with Positive Manual Assessment Findings	Lemeunier [18]	***Validity***
		Sn: 77.4% (95% CI: 64.5, 86.6)
		Sp: 83.4% (95% CI: 73.4, 90.3)
		+LR: 4.71 (95% CI: 2.75, 8.05)
		-LR: 0.27 (95% CI: 0.16, 0.45)
		**Cervical Instability**
**Diagnostic Test Studied**	**Studies (author last name and year)**	**Statistics Reported**
Canadian Cervical Spine Rules (CCR)	Moser [25]	*Physicians* Sn: 1.0 (95% CI 0.91 to 1.00) Sp: 0.43 (95% CI 0.42 to 0.45) NPV: 100% *Triage Nurses* Sn: 0.90 (95% CI 0.76 to 0.95) *ER Nurses* Sn: 1.00 Sp: 0.51 (95% CI 0.42 to 0.45) NPV: 100% *ER Physicians*: Sn: 1.0 (95% CI 0.91 to 1.0) Sp: 0.43 (95% CI 0.39 to 0.54) NPV: 100%
	Vazirizadeh-Mahabadi [49]	*EMS Personnel*
		Area under the ROC curve: 0.793 (95% CI: 0.657, 0.884)
		Pooled Sn: 0.987 (95% CI: 0.957, 0.996)
		Pooled Sp: 0.167 (95% CI: 0.073, 0.336)
		+LR: 1.184 (95% CI: 0.837, 1.675)
		-LR: 0.081 (95% CI: 0.021, 0.308)
		DOR: 14.647 (95% CI:3.678, 58.336)
NEXUS Criteria	Paykin [48]	*>65 years old*
		Sn: 0.66–1.00
	Vazirizadeh-mahabadi [49]	*EMS Personnel*
		Area under the ROC curve: 0.708 (95% CI: 0.647, 0.762)
		Pooled Sn: 0.899 (95% CI: 0.845, 0.936)
		Pooled Sp: 0.398 (95% CI: 0.315, 0.488)
		+LR 1.494 (95% CI: 1.146, 1.949)
		-LR 0.254 (95% CI: 0.155, 0.414)
		DOR: 5.894 (95% CI: 3.372, 10.305)
Sharp-Purser Test (SPT)	Mansfield [52]	*Rheumatoid Arthritis*
		Sn.19 (95% CI: 0.07, 0.37) to 1.0 (95% CI: 0.94, 1.00)
		Sp.71 (95% CI: 0.56, 0.48) to 0.98 (95% CI: 0.92, 1.00)
		+LR.66 to 22
		-LR: 0.32 to 1.14

Abbreviations: DOR – Diagnostic odds ratio; Sp – Specificity; Sn – Sensitivity; LR – likelihood ratio; PPV – Positive predictive value; NPV – Negative predictive value; NEXUS – National Emergency X-Radiography Utilization Study

### Quality assessment and risk of bias

Methodological quality was assessed by two reviewers independently (SWL and BCW) using the AMSTAR 2 (A MeaSurement Tool to Assess Systematic Reviews 2) (Table 5). A third reviewer (JAS) resolved disagreements. The AMSTAR 2 consists of sixteen items designed to appraise the quality of systematic reviews but is not intended to generate an overall point score [29]. AMSTAR 2 ratings of *High, Moderate, Low*, and *Critically Low* are generated based on the number of critical weaknesses and flaws within specific domains for each systematic review [29].

**Table 5.** AMSTAR ratings.

Study Author and Year	AMSTAR 2 Rating	Conditions Studied
Moser [25]	High	Cervical Instability after trauma
Malhotra [53]	High	Cervical Instability after trauma
Romeo [34]	High	Nonspecific neck pain
Mansfield [52]	High	Upper cervical instability
Mizer [32]	High	Degenerative joint disease
Farrell [14]	High	Nonspecific neck pain, Whiplash-associated disorder
Miranda [33]	High	Nonspecific neck pain
Lemeunier [19]	High	Nonspecific neck pain
Lemeunier [18]	High	Nonspecific neck pain
Lemeunier [20]	High	Nonspecific neck pain
Owers [47]	High	Whiplash-associated disorder
Hill [36]	High	Nonspecific neck pain/predictive value for future neck pain
Franov [40]	High	Nonspecific neck pain
Usunier [16]	High	Mechanical Neck pain
Yang [37]	High	Nonspecific/mechanical neck pain, discogenic pain
De Pauw [38]	High	Chronic/nonspecific neck pain
Abichandani [35]	High	Chronic/nonspecific neck pain
Peng [39]	High	Chronic/nonspecific neck pain
Moghaddas [42]	Moderate	Chronic/nonspecific neck pain
Manchikanti [43]	Moderate	Cervical discogenic pain
Varga [44]	Moderate	Degenerative changes/nonspecific pain
Gold [41]	Moderate	Nonspecific neck pain
Paykin [48]	Moderate	Cervical Instability after trauma
Vazirizadeh-Mahabadi [49]	Moderate	Cervical Instability after trauma
Lindenmann [45]	Low	Degenerative changes/nonspecific pain
Liao [54]	Low	Cervical Instability

Risk of bias for each systematic review was assessed using the Risk of Bias in Systematic Reviews (ROBIS) tool (Supplemental Appendix D) [30]. The ROBIS tool rates risk of bias across four domains and provides an overall rating for risk of bias in each review.

### Data synthesis and analysis

Data extracted from all systematic reviews are presented in Table 4, and Supplemental Appendices B and C. Due to the heterogeneity of the data and lack of consistent reporting across included trials, no pooling of data or meta-analyses were performed.

### Deviations from prospective protocol registration

There were two deviations from the registered protocol. First, only mechanical neck pain, whiplash-associated disorders (WAD), and cervical instability were included in this review. Data on other neck-related diagnoses, such as cervicogenic headaches and cervical radiculopathy were extracted, but due to the volume of data, those results will be published separately. Second, our search was originally completed through January 2022, but to provide the most current review, the search was updated through June 2024.

## Results

### Study selection

**Figure 1.**
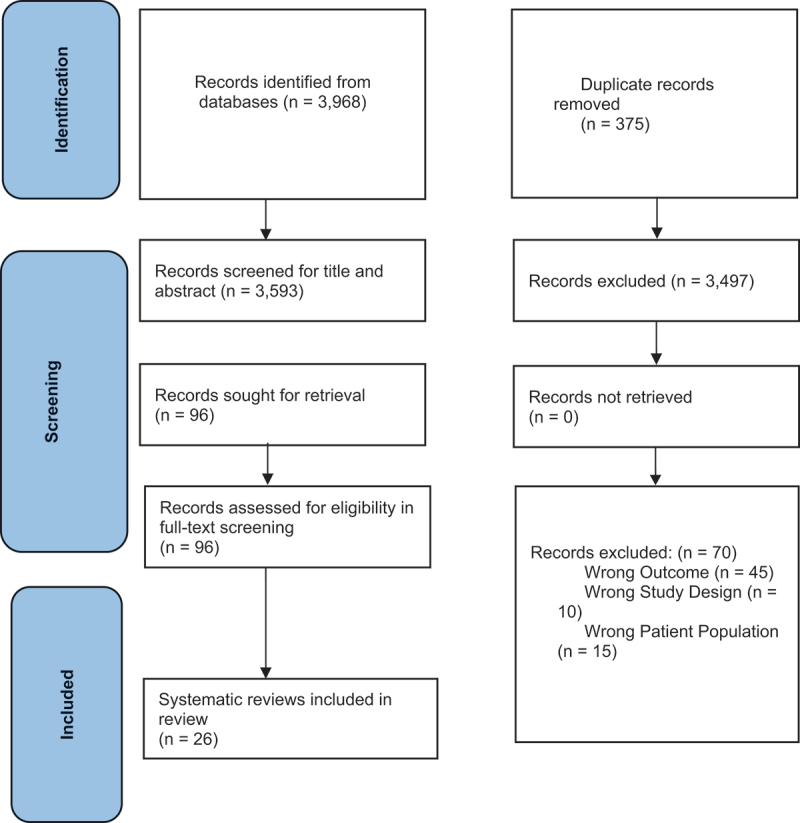
Preferred Reporting Items for Systematic Reviews and Meta-Analyses flow diagram.

The search identified 3593 articles after removing duplicates. During title and abstract screening, 3497 articles were excluded, and 70 were excluded during full-text review. The primary reason for exclusion was missing outcomes of interest. (Figure 1). After screening, 26 systematic reviews were included in this review. Cohen’s kappa for the full-text screening stage was 0.85, finding almost perfect agreement [31].

### Risk of bias of included trials

The ROBIS domain and overall ratings can be found in Supplemental Appendix D. One systematic review (3.8%) had high risk of bias, 23 (88.5%) had low risk of bias, and two (7.7%) were unclear. The most common reasons for lower ROBIS scores were unclear eligibility criteria and whether risk of bias was formally assessed in the included trials.

### Quality assessment

The results for the AMSTAR 2 appraisal are shown in Table 5. Of the 26 included systematic reviews, 18/26 were of high quality, 6/26 were of moderate quality, and 2/26 were of low quality. The most common reasons for not meeting AMSTAR 2 criteria were lack of reporting on funding sources, no justification for exclusion of articles, and lack of clarity on methodology for the literature search.

### Mechanical neck pain

#### Subjective history

Two high-quality systematic reviews [16,32] examined self-report items and subjective history to determine the likelihood and probability of diagnosing sources of NAD. Hand radiculopathy and hand numbness have good specificities (Sp = 0.89–0.92), but low sensitivities (Sn = 0.10–0.15), for diagnosing facet and uncinate joint hypertrophy [32]. However, those findings should be interpreted with caution, as neither the diagnostic odds ratio (DOR) nor positive likelihood ratios (+LR) show a statistically significant shift in the probability for diagnosing degenerative disc disease, degenerative joint disease, or uncinate hypertrophy. The DOR, sensitivity, specificity, LRs, and positive or negative predictive values (PPV or NPV) were also not significant for diagnosing degenerative disc disease, facet or uncinate joint hypertrophy based on subjective reports of headache, neck stiffness, or shoulder referral (Table 4) [32]. One review examining diagnostic accuracy of patient-reported pain location found that 36% of those with neck pain were of facet joint origin and 83% of provocative segments were correctly predicted based on pain distribution mapping when confirmed with diagnostic blocks [16].

#### Manual assessment

Two high-quality systematic reviews [16,20] examined the use of passive accessory intervertebral movement (PAIVM) testing for identifying pain originating from cervical facet joints compared to medial branch blocks [16] and facet joint blocks (Table 4) [20]. One review reported pooled validity for PAIVMs (Sn: 0.90 [95% CI: 0.85, 0.94]; Sp: 0.73 [95% CI: 0.65, 0.81]) and mechanical sensitivity using pressure pain threshold algometry (Sn: 0.88 [95% CI: 0.78, 0.95]; Sp: 0.61 [95% CI: 0.50, 0.71]) for identifying joint pain [16]. Another review examining manual assessment of joint palpation found similar sensitivity (0.89 to 0.92), but lower specificity (0.47 to 0.71) when compared to medial branch blocks [20].

#### Extension-rotation test

One high-quality systematic review [18] examined the extension-rotation test (ERT) for diagnosing facet-related neck pain, reporting a specificity of 0.59 (95% CI: 0.48, 0.70), sensitivity of 0.83 (95% CI: 0.76, 0.89), +LR of 2.01 (95% CI: 1.49, 2.72), and -LR of 0.29 (95% CI: 0.15, 0.55). See Table 4. When a positive ERT was present with PAIVM findings of joint stiffness and palpable segmental muscle tenderness, specificity increased to 0.83 (95% CI: 73.4, 90.3) and +LR increased to 4.71 (95% CI: 2.75, 8.05).

#### Cervical strength, coordination, endurance

Four high-quality systematic reviews [19,33–35] investigated neck strength, coordination, and endurance in neck pain subjects compared to healthy controls (Supplemental Appendix B). The Cranio-Cervical Flexion Test (CCFT) assesses all three components and showed significant differences (p < 0.001) in performance for neck pain subjects (24 mmHg) compared to controls (28 mmHg) [19]. One review pooled EMG and ultrasound recordings during the CCFT and discovered nonspecific neck pain subjects showed diminished activation of deep cervical flexors, but higher activation of the sternocleidomastoid and anterior scalenes across all positions compared to healthy controls [34]. The craniocervical flexion mobility at each level of activation was also reduced in those with neck pain [34]. The CCFT showed low-moderate negative correlation with pain (r = −0.29 [p > 0.05]; r = −0.37 [p = 0.02]), which is reliable and consistent with previous findings [19,34].

Chronic neck pain subjects demonstrate significant weakness with strength testing (in Newtons) for cervical flexion (SMD = −0.90 [95% CI: −1.13 to −0.67]), extension (SMD = −0.79 [95% CI: −0.99 to −0.60]), right lateral flexion (SMD = −0.74 [95% CI: −1.03 to −0.45]), and left lateral flexion (SMD = −0.75 [95% CI: −1.04 to −0.46]) compared to controls [33]. Another review found similar strength losses (in kg) in those with neck pain for flexion (MD = 3.39 [95% CI: 1.76–5.03]), extension (MD = 4.82 [95% CI: 2.93–6.71]), and lateral flexion (MD = 3.25 [95% CI: 1.75–4.76]) compared to controls [19]. Tools used to measure strength included various handheld dynamometers, a multi-cervical unit, an isokinetic dynamometer, and other unspecified dynamometer devices [19,33].

One review examined the chin tuck neck flexion test (CTNFT), neck extensor test (NET), prone neck muscle endurance test (NME), supine NME, deep cervical extensor test (DCE), and neck flexor muscle endurance test (NFME) in neck pain subjects compared to controls [19]. The CTNFT and NET have no established accuracy data, but lower scores were identified in those with neck pain compared to healthy controls [19]. The prone NME showed a weak-moderate negative correlation (r = −0.30, p = 0.01) with pain intensity. The supine NME showed a weak negative correlation (r = −0.23 [p = 0.07]) with disability via NDI. See Supplemental Appendix B.

#### Diagnostic imaging

Six high-quality systematic reviews [14,36–40], four moderate-quality systematic reviews [41–44], and one low-quality systematic review [45,46] investigated diagnostic imaging modalities for diagnosis, kinematics, and course of history for neck pain (Supplemental Appendix B). At five-year follow-up, individuals with grades 1–4 disc protrusions reported the most significant reduction in pain (MD = −2.88 [95% CI: −1.50, −4.26]) compared to those with no disc protrusion [36]. Furthermore, grades 2–4 compared to grades 0–1 (MD = −2.49 [95% CI: −0.97, −4.01]) and grades 3–4 compared to grades 0–2 (MD = −2.51 [95% CI: −0.98, −4.05]) showed significant pain reduction at five year follow up [36]. Those with mild (RR = 0.59 [95% CI: 0.36, 0.98]) and moderate-severe disc degeneration (RR = 0.46 [95% CI: 0.25 to 0.87]) showed a small but statistically significant association with reduced risk of neck pain at one year [36]. However, asymptomatic individuals with documented foraminal stenosis on MRI showed a three times greater risk of developing neck pain within 10 years (RR = 2.99 [95% CI: 1.23 to 7.23]), while development of other MRI findings was not significantly associated with neck pain at ten year follow up [36].

Modic changes in the cervical spine showed greater risk for neck pain (OR = 2.71 to 5.36), disc degeneration (OR = 2.42 to 3.90), disc protrusions (OR = 3.31 [95% CI: 1.21–9.05]; p = 0.02), and disc extrusions (RR = 2.42 [95% CI: 1.93–3.04]) [37]. However, another review reported no association between neck pain and development of Modic changes visible on MRI over a 10 year follow-up [36].

A systematic review of cross-sectional area (CSA) of posterior cervical musculature in chronic nonspecific neck pain found only rectus capitis posterior major CSA at C1-2 was significantly greater in controls compared to chronic pain (SMD = −1.18 [95% CI: −1.65, −0.71]; p < 0.001) [14]. Another systematic review found a significant reduction in longus colli CSA and anterior to posterior dimensions (APD) showing inverse relationships for CSA and disability on dominant and nondominant sides [41]. Subjects with chronic nonspecific neck pain demonstrated increased CSA of the sternocleidomastoid (SCM) and decreased CSA of deep cervical flexors and extensors compared to healthy controls [38]. One systematic review reported significantly decreased CSA of longus colli (MD = −0.23 [95% CI: −0.37, −0.08], P < 0.0001) and multiplied linear dimension (MLD) of semispinalis capitis (MD = −0.19 [95% CI: −0.34, −0.03], P = 0.32) in those with chronic neck pain, but found no significant differences for multifidus size compared to controls [39]. Additionally, those with chronic neck pain showed no differences in fatty infiltration compared to those with WAD [38], and no significant differences in disc degeneration (OR = 0.84 [95% CI: 0.57, 1.24], p = 0.39) or Modic changes (OR = 0.92 [95% CI: 0.13, 6.62], p = 0.94) in people with chronic nonspecific neck pain compared to controls [14].

One review using provocation discography found that 16–53% of chronic neck pain cases were discogenic in origin, while 41–55% were attributed to facet joints [43]. In one sample, 41% of subjects had both positive discography and facet joint blocks; another 20% had positive discography only [43]. Single-Photon Emission Computed Tomography (SPECT) imaging showed potential to detect facet arthropathy as a pain generator, but correlated with joint and soft tissue palpation findings only 12.5% of the time [44].

One systematic review used 3D motion analysis to examine posture and cervical movement during upper extremity tasks. Individuals with chronic neck pain had increased neck flexion angles during static posture assessment, reduced velocity and acceleration, and less fluidity of movement overall, but did not identify particular anatomical structures causing neck pain [42]. Another review found moderate evidence for a reduction in all acceleration variables and strong evidence for increased movement time and increased number of errors in neck pain subjects compared to controls, using optical and inertial motion capture systems, virtual reality, electromagnetic motion tracking, and head-mounted laser pointer techniques for measurement [40]. CT or MRI combined with video fluoroscopy or biplanar radiographs revealed greater ROM due to segmental instability in early cervical disc degeneration [45]. Later stages showed significant motion loss and an anterior-superior shift in the center of rotation [45]. C4 to C6 segments were shown to have the greatest contribution to mobility, but mobility decreased as degeneration progressed [45]. In those with spondylolisthesis, segmental instability increased tension throughout the surrounding musculature and ligamentous complex, limiting mobility [45].

### Whiplash-associated disorders

#### Imaging and morphology

Five high-quality systematic reviews [14,36,38,40,47] investigated imaging for morphological and kinematic changes in subjects with WAD. Techniques and findings were inconsistent across reviews. In chronic WAD subjects undergoing MRI, one review reported no significant multifidus CSA changes at C5 (p = 0.21) and C6 (p = 0.10) levels, while another reported significantly increased CSA at C5 and C6 (p < 0.01) [14,47]. Increased CSA was also reported in SCM, longus colli, longus capitis, trapezius, and splenius capitis, and cervicis muscles in another review [38]. However, the authors reported CSA increases were highly influenced by muscle fat infiltrates (MFI), with higher MFI content in cervical extensors [38]. Additional CSA findings are inconsistent between reviews, though one systematic review reported MFI to be increased in chronic WAD subjects with severe pain-related disability [14]. In whiplash injuries after motor vehicle collision, MFI presence in the cervical extensors resulted in individuals being 21 times more likely to experience a poor outcome at three months (RR = 21.00 [95% CI: 2.97 to 148.31]), but high-quality trials are scarce [36]. In acute WAD subjects, there are increased odds of muscle strains (OR = 2.69 [95% CI: 1.16, 6.21]) and vertebral body occult fractures (OR = 8.61 [95%: 1.06, 70.17]) on MRI [14].

### Cervical instability

#### Canadian cervical spine rules and NEXUS criteria

One high-quality [25] and two moderate-quality [48,49] reviews investigated the accuracy of the Canadian Cervical Spine Rules (CCR) [50] and NEXUS criteria [51]. Moser et al. (2018) found the CCR showed high sensitivity (0.90 to 1.0) and NPV (100%) when performed by physicians and nurses in various settings [25]. However, low-moderate specificity (0.43 to 0.51) increases the likelihood of false positives [25]. Another systematic review found high screening capabilities in an emergency setting for CCR and Nexus criteria with a pooled sensitivity of 0.99 (95% CI: 0.957, 0.996) and 0.90 (95% CI: 0.845, 0.936) respectively [49]. The CCR showed superior screening accuracy (-LR: 0.081 [95% CI: 0.021, 0.308]; DOR: 14.647 [95% CI: 3.678, 58.336]) compared to the NEXUS criteria (-LR 0.254 (95% CI: 0.155, 0.414); DOR: 5.894 (95% CI: 3.372, 10.305) [49].

#### Sharp-Purser test

One high-quality systematic review [52] investigated the Sharp-Purser Test’s (SPT) validity. In individuals with rheumatoid arthritis (RA), sensitivity was highly variable (0.19 [95% CI: 0.07, 0.37] to 1.0 [95% CI: 0.94, 1.00]), but specificity was more consistent (0.71 [95% CI: 0.56, 0.84] to 0.98 [95% CI: 0.92, 1.00]) [52]. Likelihood ratios also varied, with +LR ranging from 0.66 to 22.00, and -LR from 0.32 to 1.14 [52].

#### Diagnostic imaging

Two systematic reviews, one high-quality [53], and one low-quality [54], investigated diagnostic imaging for cervical instability. Ligamentous injuries were the most commonly detected injuries by MRI after blunt trauma injuries [53]. Only 16 injuries from 5,286 patients were considered unstable, but current definitions of clinically significant or unstable injuries are heterogeneous, thus, clinical applicability is unclear [53]. Another review reported MRI combined with static and dynamic radiography is necessary to determine discoligamentous injuries for C2-3 relating to fractures of the axis ring to provide a more comprehensive view of the discoligamentous complex and bony alignment, potentially indicating craniocervical dislocations [54,55]. Subjective reports of cervical hyperflexion or hyperextension are common with discoligamentous injuries and axis ring fractures [54].

Odontoid fractures may be assessed with plain radiographs or computerized tomography (CT) [53,54]. Displacement of more than 2 mm on lateral extension-flexion x-ray was a determinant for odontoid fracture, while displacement of more than 6 mm indicated instability with CT, despite widely accepted criteria of 5 mm displacement or angulation of more than 11 degrees [53,54]. Occipital condyle fractures are difficult to detect with plain radiographs, thus, CT is more appropriate to determine instability [54].

## Discussion

This overview of systematic reviews included 26 systematic reviews with the purpose of providing an updated synthesis of diagnostic utility of clinical features, tests, and measures for NAD. The results were highly heterogeneous for subject populations, clinical features examined, and aims of each systematic review. However, several findings emerged.

### Mechanical neck pain

For mechanical neck pain, diagnostic injections, manual assessment, and ERT provide moderate to high diagnostic capability for diagnosing facetogenic pain, while subjective reports can aid in diagnosing facet and uncinate joint hypertrophy [16]. Facetogenic pain, can be defined as pain arising from any structural component of the facet joints, including the capsule, synovial membrane, hyaline cartilage, and bone, while facet joint hypertrophy is commonly associated with arthritic changes, resulting in hypertrophy of the articular processes, synovial cysts [56].

Diagnostic injections are the current reference standard for diagnosing facetogenic pain, but there have been calls for more conservative, less invasive testing methods to improve clinical diagnosis [16]. Moderate correlation has been shown for patient-reported facet joint referral pain mapping and diagnostic injections for diagnosing facetogenic pain [16]. Manual testing can also be reliable and useful for diagnosing facetogenic pain, with comparable results to diagnostic injections when combined with sensitivity to palpation of paraspinal muscles of the same segment, and greater specificity when combined with a positive ERT [16,18]. Patient reports of hand radiculopathy and hand numbness have strong specificity but low sensitivity for diagnosis of facet joint hypertrophy and uncinate joint hypertrophy [32]. These findings may aid clinicians in diagnosing facet-related pain using joint mobility assessment, soft tissue palpation, ERT, and subjective reports. Manual assessment, such as soft tissue palpation and cervical joint mobility, demonstrate inconsistent inter-rater reliability when performed alone and should be interpreted with caution [20]. The strongest reliability exists when pain and joint stiffness are both present during PAIVMs [18,20].

Results from this overview of systematic reviews show a reduction in neck pain as neck strength and control improves [19,33–35]. Neck endurance, strength, and motor control tests have shown preliminary validity and reliability for assessing subjects with neck pain both with and without radiating symptoms [19,33–35]. However, there was no data suggesting neck strength, endurance, and motor control tests implicate specific structures as causative factors for people with NAD.

Imaging has been associated with identifying diagnoses related to structural changes that some consider to help determine pain locations and predict pain; however, recent research questions this [57–59]. There are structural and morphological changes seen on imaging in those with and without neck pain [21–23,26,40]. MRI findings for high-grade disc protrusion and all ranges of disc degeneration are associated with a reduction in risk of neck pain long term, while findings of foraminal stenosis demonstrated a greater risk for developing neck pain long term. MRI findings are inconsistent in predicting pain related to CSA, MFI, or Modic changes, and implicating specific structures. Subjects with mechanical neck pain, WAD, and healthy controls have notable overlap in abnormal imaging findings [14,36–38,41,58]. Current data does not support a causal relationship between structural changes on imaging, clinical test results, and symptoms; it is unclear if MRI findings predict future neck pain [14,36–38,41,58].

### Whiplash-associated disorders

Morphological changes of CSA and MFI may be present on MRI in individuals with WAD; however, findings are inconsistent and there is insufficient evidence that MFI or CSA changes contribute to neck pain [14,36,38,47]. Previous data suggests conflicting findings regarding CSA in lower cervical spine musculature [38]. Inflammation may contribute to increased CSA in acute WAD; MFI may contribute to CSA increases in chronic WAD cases [47]. Clinicians should be cautious when interpreting morphological changes due to overlapping features with other neck pain diagnoses [15,47]. A thorough subjective history should be performed to determine a mechanism of injury, such as a fall or motor vehicle accident, and aid in ruling out other potential diagnoses.

### Cervical instability

The SPT has poor reliability and diagnostic accuracy of cervical instability, with sparse evidence for assessment in high-risk populations [52]. The SPT should not be used in isolation and should be interpreted with consideration of additional clinical findings when diagnosing cervical instability. There is strong evidence that the need for imaging in cervical instability can be accurately ruled out by physicians, nurses, and paramedics, using the CCR and NEXUS criteria, with the CCR having greater screening power [25,48,49]. Reliability for the specificity of these clinical prediction rules is variable, increasing the risk for false positives [60].

Occipital condyle fractures are difficult to detect using plain radiography alone, as the fractures are often not visible [61–64]. CT scan is more appropriate than plain radiographs for the detection of odontoid or occipital condyle fractures and misalignments in the upper cervical spine due to greater sensitivity and reliability [54,62]. Furthermore, MRI combined with static and dynamic radiography is considered necessary to determine the integrity of the disco-ligamentous complex of the second and third cervical vertebrae, as it provides comprehensive information on ligamentous injury and bony alignment [54,55]. Plain radiographs have been shown to miss ligamentous disruption and craniocervical dislocations when performed after trauma [55]. No evidence included in this review compared relationships or accuracy of movement-based clinical tests to imaging findings to improve clinical diagnosis of cervical instability.

### Potential limitations

There are multiple potential limitations to this overview of systematic reviews. First, the data collected were broad and heterogeneous, limiting the ability to perform meta-analyses. Second, due to the heterogeneity of NAD presentations, several reviews included ‘non-specific neck pain’ but did not attempt to relate clinical features with a specific mechanical cause. Third, due to the nature of overviews of systematic reviews, it is possible that primary studies were included in multiple systematic reviews included in this overview of systematic reviews, which may result in overrepresentation. Fourth, original trial data from the randomized trials within the included systematic reviews was not directly assessed. Fifth, systematic review quality was assessed for the included systematic review as a whole. However, no judgments regarding the quality of individual trials within each systematic review can be made. Sixth, this review did not find any systematic reviews investigating psychosocial variable influences on NAD. Due to the multifactorial nature of pain and varying clinical presentations, specific mechanical diagnoses may be complicated by psychosocial influences and should be investigated [5,65].

## Conclusion

Recent evidence examining clinical features for the diagnosis of mechanical causes of NAD is unclear. Diagnostic imaging is inconsistent at predicting neck pain or dysfunction, with abnormalities often present in both pathologic subjects and healthy controls. Overlap in diagnostic imaging findings with various pathologies exists, limiting the ability to accurately diagnose individuals with NAD due to specific mechanical causes, such as joint, disc, and muscular dysfunction. However, imaging does have good validity in the diagnosis of traumatic structural instability. For individuals with mechanical neck pain, hand radiculopathy and hand numbness have strong specificity but low sensitivity for diagnosing degenerative changes. Positive manual assessment findings and ERT provide moderate to high diagnostic validity for indicating facetogenic pain and may be comparable to the validity of diagnostic injections. The CCR and NEXUS criteria can be used to confidently rule out the need for imaging, but the SPT has limited utility for diagnosing cervical instability in isolation. Future research should seek to improve diagnostic accuracy of clinical testing for differentiation of common neck pain diagnoses due to frequent overlap of presentation for multiple pathologies.

## Supplementary Material

Supplemental Appendix C_Neck Pain Narrative Summaries.docx

Supplemental Appendix A_Search Strategies and Results.docx

Supplemental Appendix D_ROBIS Table.docx

Supplemental Appendix B_Neck Pain Clinical Features Associations and Risk Factors.docx

## Data Availability

The data supporting the findings of this review are available with the article and supplemental materials. Data may also be found at Mendeley Data with the following citation: Subialka, Joshua; McConnell, Ryan; Williams, Brandon; Lowe, Scott (2023), ‘An overview of systematic reviews investigating the accuracy, reliability, and relationships of tests and measures for diagnosis of neck pain’, Mendeley Data, V1, doi: 10.17632/85nd9krcdx.1

## Previous iterations

Data from this manuscript were previously showcased in a poster presentation at the 2023 American Academy of Orthopaedic Manual Physical Therapists’ Conference. The poster presentation information can be accessed at: https://works.bepress.com/scott-lowe/4/.
