# Supplementary material for: An overview of systematic reviews investigating clinical features for diagnosing neck pain and its associated disorders
Source: J Man Manip Ther. 2024 Dec 13;33(4):286–98. doi: 10.1080/10669817.2024.2436403 (PMC12281667; doi:10.1080/10669817.2024.2436403)
Supplement: Supplemental Appendix C_Neck Pain Narrative Summaries.docx [file YJMT_A_2436403_SM6022.docx]

**SUPPLEMENTAL APPENDIX C**

| **Mechanical Neck Pain** | | |
| --- | --- | --- |
| **Topic Studied** | **First Author and Year** | **Summary of Relevant Findings** |
| Self-Report Items with Subjective History | Mizer 2017^32^ | Statistics on subjective examination findings compared to reference standards of cervical radiographs. Overall, individual subjective questions show minimal diagnostic value in identifying neck pain conditions without physical examination findings. |
| Posture | Lemeunier 2018^20^ | Reliability:  **Absolute reliability between the three examiners was 1.8 degrees  Excessive or diminished thoracic kyphosis cannot be reliability assessed via visual inspection due to highly variable inter-rater reliability.  Excessive scapular protraction can be assessed reliably with visual inspection.  Inter-rater reliability of manual palpation with segmental mobility assessment of C2-7 is unreliable, though tenderness over C1 transverse process has greater inter-rater reliability.  Validity:  Inconsistent evidence for validity of CROM device, digital caliper, or goniometer to discern differences in head posture between patients with NAD and healthy subjects.  Additionally, correlation between craniovertebral angle and neck pain intensity in patients with NAD was not statistically significant, however, forward head (upper thoracic and craniovertebral) posture was greater than asymptomatic age-matched controls and associated with an increase in perceived disability.  No specific statistics reported for validity of cervicothoracic posture assessment. |
| Manual Assessment | Usunier 2018^16^ | The results of this study suggest that skilled clinicians may be able to diagnose pain of facetogenic origin using manual application of intersegmental joint pressures. No other tests were identified that could be confidently endorsed  Heterogeneity may be due to the difference in collection methods (digital algometer using interval-level scaling vs. manual pressure using nominal-level scaling |
|  |  |  |
|  | Lemeunier 2018^20^ | Patients with neck or neck -related pain had more trigger points identified by static palpation in the temporalis, upper trapezius, sternocleidomastoid, levator scapula, scalenes, and suboccipital muscles compared to healthy controls. The number of active trigger points was also positively correlated with pain intensity, but negatively correlated with CROM and PPT at C5-6.  Presence of a trigger point in the upper trapezius is correlated with ipsilateral cervical dysfunction at C3 and C4 joints (p<0.03) but strength of the correlation is unknown.  Specificity for joint mobility assessment compared to nerve blockades increased to 0.75 when joint palpation was combined with tenderness at the same cervical paraspinal muscle segment.  Reliability of manual joint palpation is inconsistent across studies for C2-7 levels, but can be assessed with less measurement error for C1 transverse process static palpation (k=0.83 [95% CI: 0.74-0.92). Soft tissue palpation to assess tender points in cervical muscles is also inconsistent across two studies both within and between raters.  Extension-rotation test (ERT) +LR and specificity can be improved by combining with manual examination for manual spinal examination (MSE) and palpation for segmental tenderness (PST) of muscles overlying facet joints. -LR and sensitivity are minimally changed with extension-rotation test alone compared to ER+MSE, ER+PST, and ER+MSE+PST. |
| Cervical joint referral patterns, or ‘‘pain maps” | Usunier 2018^16^ | Two studies showed some relationship between pain mapping and results confirmed with diagnostic blockade, however, neither provided sufficient evidence for statistical pooling. |
| Neck Functional Tests | Lemeunier 2020^19^ | Subjects with neck pain demonstrated shoulder elevation weakness and grip strength weakness compared to controls. Hand grip weakness was significantly associated with increased levels of pain, disability, anxiety, and depression, as well as decreased functional, though correlations for each reported outcome were weak. |
| Cervical Strength | Lemeunier 2020^19^ | CCFT showed a negative correlation with pain and disability, with significance levels varying between two studies.  Neck pain subjects have a significant decrease (p<0.001) in CCFT strength (24 mmHg) compared to healthy controls (28 mmHg).  Cervical muscle strength was tested via handheld dynamometer. |
|  | Romeo 2022^34^ | Pooled EMG and US recordings during the CCFT for non-specific neck pain showed decreased activation of deep cervical flexors, higher activation levels of the sternocleidomastoid and anterior scalene, and reduced craniocervical flexion motion muscle across all positions compared to asymptomatic subjects. |
|  | Abichandani 2023^35^ | All measures used in this study showed moderate to high reliability for all testing. However; it also demonstrates a lack of a gold standard regarding the most appropriate measure for neck strength due to low quality of evidence and grossly inadequate risk of bias assessments. |
| Cervical Endurance | Lemeunier 2020^19^ | Clinical accuracy of neck muscle endurance tests are not known, though preliminary evidence suggests there is validity for neck endurance testing in some patients with neck pain.  Significance for validity of chin tuck neck flexion test and NET not reported in tables or write up.  Neck pain subjects with radiating upper extremity symptoms show significantly reduced NME times compared to healthy controls (prone: p < 0.01; supine: p = 0.017).  Prone NME times are negatively correlated with pain intensity.  Supine NME times are negatively correlated with disability via the NDI.  There was a negative correlation of DCE test times compared with pain and disability, and a positive correlation between DCE test times and SF-36-PCS scores. |
| Magnetic Resonance Imaging (MRI) | Hill 2018^36^ | It is unclear if MRI findings are predictive of future neck pain. Due to heterogeneity, it was not possible to pool outcomes.  Subjects with higher grades of disc protrusion at baseline reported lower levels of neck pain at 1 and 5 year follow ups compared to those with lower grades or no disc protrusion on MRI. |
|  | Yang 2020^37^ | Modic changes in cervical spine show positive correlation with neck pain and disc degeneration. This ranges from 5-40%; 5% was reported in asymptomatic populations, while 40% was reported for neck pain populations. Type II Modic changes are more prominent and C5-6 is the most common level. |
|  | Farrell 2019^14^ | No changes in CSA of multifidus at C2-3, semispinalis capitis at C2-3, and SCM at C2-3 and C5-6 between chronic NSNP and controls, but quality of evidence was very low.  Low quality of evidence found no differences in CSA of longus capitis/colli at C2-3 between chronic NSNP and controls.  Moderate quality evidence shows greater CSA of rectus capitis posterior major found at C1-2 in controls compared to NSNP subjects, but no difference in CSA for rectus capitis posterior minor at C1-2.  One study found smaller CSA of semispinalis cervicis at C5-6 in females with chronic NSNP compared with controls.  One study found greater spinal cord compression in chronic NSNP subjects compared to controls.  In NSNP vs controls, no between-group differences were noted for facet joint MRI findings or other postural abnormalities. |
| Discography | Manchikanti 2018^43^ | The available literature on cervical discography continues to be of low quality (level IV). |
| Motion Analysis | Moghaddas 2019^42^ | **Cervical Kinematics Chronic Neck Pain vs. Controls - 3D Motion Analysis**  Significantly reduced trunk rotation while walking with head rotated.  Significant reduction in average neck velocity and acceleration during overhead reaching  Lower peak and mean velocities and impaired motion smoothness  Increased head/neck flexion and reduced lateral flexion and rotation angles |
|  | Franov 2022^40^ | Measurements in this study were electromagnetic motion tracking, optical motion capture systems, virtual reality tracking systems, and inertial motion capture systems. Unclear reporting on which techniques were used to capture specific variables.  ***Head Kinematics in Neck Pain vs. Controls***  *Velocity Variables*  Conflicting evidence with studies reporting either decreased or no differences in peak velocity.  *Acceleration Variables*  Reduced mean acceleration - limited level of evidence.  Conflicting evidence on peak acceleration  Decreased magnitude of circumduction vectors - very limited evidence  All Variables - unconstrained movement tasks were reduced - moderate evidence  *Temporal Variables*  Increased movement time - strong level of evidence  No differences in reaction time - very limited evidence  Increased deceleration phase - limited evidence  No differences in acceleration phase - limited evidence  Conflicting level of evidence for ratio of phase duration  *Movement Smoothness*  Increased spectral entropy - limited evidence  Increased speed index of deviation - limited evidence  Increased number of jerk peaks - very limited evidence  Conflicting evidence for normalized jerk cost and number of velocity peaks  *Movement Accuracy*  Increased number of errors - strong level of evidence  Decreased time on target - limited level of evidence  Conflicting evidence for point deviation |
| Single-Photon Emission Computed Tomography Imaging (SPECT) | Varga 2023^44^ | SPECT identified potential pain generators in 92% of cervical spine scans. For the cervical and lumbar spine regions combined, the scan localized SPECT-positive facet joint targets in 65% of the referral population. However, the correlation with positive findings on the clinical exam was poor. From 25 cervical scans, 52% found facet arthropathy and 36% showed disc degeneration. Localized tenderness was related to SPECT-positive facet arthropathy in only 1 of 8 cases, and clinically suspected facet arthropathy correlated with positive SPECT findings in only 8/21 (38%) of patients.  There were no significant differences between groups for SPECT+ and SPECT- findings using targeted injections in both immediate and 2 week follow ups. For subjects with a previous failed facet injection, adding SPECT/CT-guided injections reduced pain at two weeks (p = 0.02) |
| Multiple Imaging Modalities | Gold 2017^41^ | Measurements used in this study were MRI, US, far infrared thermography, near infrared spectroscopy (NIRS), and laser Doppler flowmetry. Unclear reporting on which techniques were used to capture specific variables.  **Quantitative Imaging Biomarkers for Neck Pain**  Decreased neck muscle size   - - Longus colli   - Semispinalis capitis   - Semispinalis cervicis   - Cervical multifidus C3-C6   Reduced trapezius blood flow, relative blood volume, and oxygen saturation at rest and in response to UE tasks in subjects with neck and shoulder pain.  No significant difference in fat index indicating fatty infiltration in those with neck pain vs controls.  In subjects with neck and shoulder pain, trapezius metabolism, as indicated by minimal and maximal standardized 18F-FDG uptake values, was inversely correlated with pain. However, this was only found in one study with a small sample size. |
|  | De Pauw 2016^38^ | *Measurements used in this study were MRI and US*  **Morphologic Changes in Chronic Neck Pain**.  **Idiopathic Chronic Neck Pain**  *Cervical Flexor Musculature*  Increased CSA of:   - Sternocleidomastoid   Decreased CSA of:   - Longus Colli - Longus Capitis   *Cervical Extensor Musculature*  Decreased CSA of:   - Rectus capitis major - Rectus capitis minor - Multifidus - Semispinalis cervicis - Semispinalis capitis - Splenius capitis   *Fatty Infiltration*  Current research has only found fatty infiltration in WAD, not chronic idiopathic neck pain  Summary:  Moderate evidence showing higher CSA changes in all flexor muscles except deep flexor muscles in idiopathic chronic neck patients. Increased CSA in extensor muscles at upper cervical levels in WAD patients. Majority of suboccipital changes were due to fatty infiltrations. Chronic neck pain shows decreased CSA in almost all extensor muscles. |
|  | Lindenmann 2022^45^ | Measurements used in this study were lateral or dual neck radiographs, fluoroscopy, computed tomography (CT), magnetic resonance imaging (MRI),or combinations of the above. Unclear reporting on which techniques were used to capture specific variables.  ***Cervical Disc Degeneration vs. Controls***  *Flexion/Extension ROM*  Grades I and 2 degeneration were associated with 2-3 times higher instability compared to 3 and 4. With more instability comes more laxity of the ligaments around the facet joints. This leads to increased osteophyte formation, thus limiting segment mobility. The initial stages of degeneration increase ROM due to instability; while the later stages are defined as loss of motion.  *Angular and Translational ROM*  The greatest variation was seen in mild to moderate degeneration; while more severe degeneration restricts segments to a point of near immobility.   - C3/4 had more mobility in severe degeneration rather than early. - C5/6 and C/7 were 15-20% more mobile in moderate and severe degeneration compared to early stages. - Progression from moderate to severe degeneration restriction   - C5/6: 36%   - C6/7: 7% - C4-C6 is responsible for having the greatest contribution to mobility that decreased proportionally to the degree of degeneration.   *Center of Rotation*  The mean instant center of rotation shifts anterior and higher in moderate to severe degeneration. Decreased disc height correlates with anterior-superior translation.  ***Spondylolisthesis vs Controls***  Most affected levels were C4/5 and C5/6 due to higher levels of mobility.  The listhesis level has the highest level of degeneration while the cephalad level has the lowest. When a vertebra is displaced, muscle spasm and increased ligamentous tension is more common and can lead to impaired mobility patterns. Current evidence suggests this hypomobile pattern affects the dysfunctional segment as well as adjacent segments above and below.  Overall:  Restricted mobility of adjacent segments could potentially help differentiate spondylolisthesis from severe degeneration when using dynamic imaging. This is because severe degeneration of adjacent structures would compensate for the loss of mobility. Differentiation would be more challenging if both spondylolisthesis and severe degeneration were present. |
|  | Peng 2022^39^ | Measurements used in this study were MRI and US.  In those with chronic non-specific neck pain the longus colli and semispinalis capitis muscle sizes were slightly smaller; however; no differences in multifidus size compared to controls. |

Abbreviations: NAD - Neck associated disorders; MD - Mean difference; PPT - Pressure pain threshold; NET - Neck extensor test; NME - Neck muscle endurance test; DCE - Deep cervical extensor test; CCFT - Craniocervical flexion test; CSA - Cross sectional area; NDI - Neck disability index; NSNP - Nonspecific neck pain

| **Whiplash Associated Disorders (WAD)** | | |
| --- | --- | --- |
| **Diagnostic Test Studied** | **Studies (author last name and year)** | **Other Qualitative Data Reported** |
| Morphological changes | Farrell 2019^14^ | **Acute WAD (MRI)**  Two studies reported no difference in MRI findings between acute WAD and controls.  One study found posterior disc protrusion occurred more frequently in the WAD while loss of disc height and anterior protrusion was more prevalent in controls.  No changes in facet joints were noted between acute WAD and healthy control groups.  Transverse ligament thickness occurs more frequently in acute WAD compared to controls.  CSA findings in acute WAD for cervical multifidus, semispinalis cervicis, deep and total neck extensor muscles, and sternocleidomastoid show inconsistent results.  **Chronic WAD (MRI)**  There were no differences in CSA of multifidus at the C4-C6  levels between the chronic WAD and control groups (two  studies  All other showed no significant difference: multifidus CSA, alar ligament signal intensity in chronic WAD, muscle CSA, disc degeneration, modic changes in chronic NSNP. However, the authors did note that the confidence intervals were relatively wide and meaningful associations could exist, just not for meta analysis. High heterogeneity may reflect this  Rectus capitis posterior major had less cross sectional area in chronic WAD compared to control with a large effect size with SMD of -1.18.  Due to low quality of evidence, conclusions cannot be made on MRI findings and relationships to WAD or nonspecific neck pain vs control groups. |
|  | Owers 2018^47^ | **MRI**  The strength of the evidence for cervical muscle morphometric changes on MRI after whiplash is inconsistent for CSA and MFI. There is not enough evidence that MFI is an accurate indication of neck pain in WAD subjects. Increase in CSA may be due to inflammation in acute WAD, or MFI in chronic WAD. |
|  | De Pauw 2016^38^ | **Whiplash Associated Disorder (MRI and US)**  *Cervical Flexor Musculature*  Increased CSA of   - Sternocleidomastoid - Longus Colli - Longus Capitis   * These changes were highly influenced by fatty infiltration  *Cervical Extensor Musculature*  Increased CSA of higher levels:   - Trapezius - Splenius capitis - Splenius cervicis   *The same muscles have been shown to have normal to decreased CSA of lower levels.  *Fatty Infiltration*  Higher amount in cervical extensors except multifidus and was found in deep cervical flexors. |
| Motion Analysis | Franov 2022^40^ | Measurements in this study were electromagnetic motion tracking, optical motion capture systems, virtual reality tracking systems, and inertial motion capture systems.  ***Head Kinematics in WAD vs. Controls***  Electromagnetic motion tracking, optical motion capture, virtual reality tracking, and inertial motion capture were used to assess head kinematics.  *Velocity Variables*  Decrease in mean velocity - moderate level of evidence  *Acceleration Variables*  Decrease in peak acceleration - limited evidence  *Temporal Variables*  Conflicting level of evidence for movement time  Increased reaction time - moderate level of evidence  *Movement Smoothness*  Increased root mean square velocity - limited evidence  *Movement Accuracy*  Increased number of errors - strong level of evidence  Increased point deviation - moderate level of evidence  Decreased time on target - moderate level evidence |

Abbreviations: CSA - Cross sectional area; SMD - Standard mean difference; CROM - Cervical range of motion; MFI - Muscle fat infiltration; NSNP - Nonspecific neck pain

| **Cervical Instability** | | |
| --- | --- | --- |
| **Diagnostic Test Studied** | **Studies (author last name and year)** | **Other Qualitative Data Reported** |
| Canadian Cervical Spine Rules (CCR) | Moser 2018^25^ | CCR shows high sensitivity and NPV, suggesting it is highly informative for ruling out need for cervical spine radiographs.  CCR shows low to moderate specificity, resulting in an important number of false positives.  Modified CCR version: criterion for “delayed onset of neck pain” was removed. |
| CCR and Nexus Criteria | Vazirizadeh -Mahabadi 2023^49^ | There is good evidence that the need for imaging in cervical instability can be accurately ruled out by using the CCR and NEXUS criteria, with the CCR having greater screening power |
| Sharp-Purser Test (SPT) | Mansfield 2020^52^ | Minimal to no increase to large shift in posttest probability with a positive SPT  Small to no effect on posttest probability with a negative SPT.  Reliability is poor, which limits use as a screening test outside of RA population.  Evidence is scarce supporting or refuting performing SPT in high risk populations. |
| Magnetic Resonance Imaging (MRI) | Malhotra 2017^53^ | Ligamentous injuries most commonly detected after blunt traumatic injury, but only 16 injuries from 5,286 patients were considered unstable. However, literature is heterogenous for outcomes defined as clinically significant or unstable injury. |
|  |  |  |
|  |  |  |
|  |  |  |
|  |  |  |
|  |  |  |
|  |  |  |
|  | Liao 2020^54^ | MRI necessary to determine integrity of discoligamentous injuries for C2-3 relating to fractures of the axis ring. Subjective report of trauma, particularly hyperflexion or hyperextension injury. |
| X-ray | Liao 2020^54^ | Occipital condyle fractures hard to detect using x-ray  Dynamic sagittal displacement of more than 2 mm on lateral extension-flexion x-ray is determinant for odontoid fracture. |
| Computerized tomography (CT) | Liao 2020^54^ | Indicated for odontoid fracture  Displacement of more than 6 mm indicated instability despite widely accepted criteria of 5 mm displacement or angulation more than 11 degrees |
|  |  |  |

Abbreviations: RA - rheumatoid arthritis; NPV - negative predictive value
