# Supplementary material for: An overview of systematic reviews investigating clinical features for diagnosing neck pain and its associated disorders
Source: J Man Manip Ther. 2024 Dec 13;33(4):286–98. doi: 10.1080/10669817.2024.2436403 (PMC12281667; doi:10.1080/10669817.2024.2436403)
Supplement: Supplemental Appendix A_Search Strategies and Results.docx [file YJMT_A_2436403_SM6021.docx]

**SUPPLEMENTAL APPENDIX A**

Initial date of conducted searches: 1/13/22

Embase (Elsevier)

| 1. | 'neck pain'/exp OR 'brachial plexus neuropathy'/exp OR 'neck injury'/exp OR 'thorax outlet syndrome'/exp OR 'thorax outlet syndrome'/exp OR 'torticollis'/exp OR 'brachial plexus neuropathy'/exp OR 'brachial plexus neuropathy'/exp OR 'myofascial pain'/exp OR 'spondylosis'/exp OR (neckache OR neckaches OR neckpain OR neckpains OR whiplash OR whiplashes OR whiplashing OR whiplashed OR cervicodynia$ OR cervicalgia$ OR brachialgia$ OR torticollis OR 'myofascial pain syndrome' OR 'myofascial pain syndromes' OR 'thoracic outlet syndrome' OR 'thoracic outlet syndromes' OR 'thorax outlet syndrome' OR 'spinal osteophytosis'):ti,ab | 70,974 |
| --- | --- | --- |
| 2. | 'neck'/exp OR 'neck muscle'/exp OR 'cervical plexus'/exp OR 'spinal nerve'/exp OR 'atlantoaxial joint'/exp OR 'atlantooccipital joint'/exp OR 'first cervical vertebra'/exp OR 'brachial plexus'/exp OR 'second cervical vertebra'/exp OR 'odontoid process'/exp OR 'thoracic spine'/exp OR 'cervical spine'/exp OR 'intervertebral disk'/exp OR (neck OR necks OR C1 OR C2 OR C3 OR C4 OR C5 OR C6 OR C7 OR T1 OR T2 OR T3 OR T4 OR T5 OR T6 OR T7 OR T8 OR T9 OR T10 OR T11 OR T12 OR odontoid OR odontoids OR occipital OR occipitalis OR occipitali$ation OR occipitali$ed OR occipito$cervical OR occipito$temporal OR atlanto OR atlanto$cervical OR atlanto$occipital OR atlanto$axial OR atlanto$dental OR brachial OR cervico$brachial OR cervico OR ((thoracic OR cervical) NEAR/3 (vertebra OR vertebrae OR vertebraes OR vertebral OR spine OR spines OR spinal OR outlet OR outlets OR lumbar OR lumbral OR disk OR disks OR disc OR discs)) OR trapezius OR cervicogenic OR cervico$facial OR cervico$thoracic OR cervico$vertebral OR cervico$spinal OR cervical$brachial OR cranio$vertebral):ti,ab | 1,157,490 |
| 3. | 'gynecologic disease'/exp OR 'uterus'/exp OR (genital disease* OR uterus OR uterine):ti,ab | 926,561 |
| 4. | #2 NOT #3 | 1,135,568 |
| 5. | 'spine disease'/exp OR (headache OR headaches OR pain OR pains OR pained OR painful* OR ache OR aches OR ached OR aching OR achy OR achiness sore OR soreness OR stiff OR stiffness OR discomfort OR discomforts OR uncomfortable* OR injury OR injuries OR injure OR injured OR injuring OR sprain OR sprains OR sprained OR strain OR strains OR strained OR monoradiculopathy OR monoradiculopathies OR mononeuropathy OR mononeuropathies OR radiculopathy OR radiculopathies OR polyradiculopathy OR polyradiculopathies OR neuritis OR neuropathy OR neuropathies OR neuropathic OR radiculitis OR (temporomandibular NEAR/2 disorder$) OR arthriti$ OR osteoarthriti$ OR fibromyalgia$ OR spondylosis OR spondylitis OR spondylolisthesis OR spondylosis OR spondylolysis OR spondylolyses OR spondylitis OR spondylolisthesis OR herniation$ OR herniate OR herniates OR herniated OR herniating OR slipped OR prolapse OR prolapses OR prolapsed OR prolapsing OR displace OR displaces OR displaced OR displacement$ OR bulg* OR osteophytosis OR discitis OR diskitis OR discopathy OR discopathies OR displacement$ OR degeneration$ OR degenerated OR degenerating OR neuralgia OR neuralgias):ti,ab | 3,321,474 |
| 6. | #4 AND #5 | 251,922 |
| 7. | #1 OR #6 | 301,959 |
| 8. | di.fs OR 'diagnosis'/exp OR 'sensitivity and specificity'/exp OR 'predictive value'/exp OR (sensitiv* OR specificit* or diagnos* OR detect* OR screening$ OR screen$ OR screened OR 'predictive value' OR 'predictive values'):ti,ab | 13,135,561 |
| 9. | #7 AND #8 | 166,922 |
| 10. | ('meta analysis'/exp OR 'systematic review'/exp OR 'meta analysis (topic)'/exp OR 'systematic review topic'/exp) OR (systematic* NEAR/3 review*):ti,ab OR (systematic* NEAR/3 overview*):ti,ab OR (methodologic* NEAR/3 review*):ti,ab OR (methodologic* NEAR/3 overview*):ti,ab OR (quantitative NEAR/3 review*):ti,ab OR (quantitative NEAR/3 synthes*):ti,ab OR ('meta regression*' OR metaregression* OR meta-analy* OR metaanaly* OR 'systematic review' OR 'systematic reviews' OR cochrane OR meta-analysis):ti,ab | 625,756 |
| 11. | #9 AND #10 | 2,788 |
| 12. | #11 AND [2016-2022]/py NOT ([conference abstract]/lim OR 'conference abstract'/exp OR 'conference abstract'/it) | 1,231 |

NLM MEDLINE

Translated from OVID MEDLINE

| 1.  Neck pain | Neck Pain[mesh:noexp] OR Brachial Plexus Neuropathies[mesh] OR neck injuries[mesh] OR whiplash injuries[mesh] OR thoracic outlet syndrome[mesh:noexp] OR cervical rib syndrome[mesh:noexp] OR Torticollis[mesh:noexp] OR brachial plexus neuropathies[mesh] OR brachial plexus neuritis[mesh] OR myofascial pain syndromes[mesh:noexp] OR Spinal Osteophytosis[mesh] OR neckache[tiab] OR neckaches[tiab] OR neckpain[tiab] OR neckpains[tiab] OR whiplash[tiab] OR whiplashes[tiab] OR whiplashing[tiab] OR whiplashed[tiab] OR cervicodynia[tiab] OR cervicalgia[tiab] OR brachialgia[tiab] OR torticollis[tiab] OR "myofascial pain syndrome"[tiab] OR "myofascial pain syndromes"[tiab] OR "thoracic outlet syndrome"[tiab] OR "thoracic outlet syndromes"[tiab] OR "thorax outlet syndrome"[tiab] OR "spinal osteophytosis"[tiab] | 34,073 |
| --- | --- | --- |
| 2.  Neck | neck[mesh] OR neck muscles[mesh:noexp] OR cervical plexus[mesh] OR atlanto-axial joint[mesh:noexp] OR atlanto-occipital joint[mesh:noexp] OR Cervical Atlas[mesh:noexp] OR spinal nerve roots[mesh:noexp] OR brachial plexus[mesh] OR axis, cervical vertebra[mesh:noexp] OR odontoid process[mesh:noexp] OR Thoracic Vertebrae[mesh:noexp] OR cervical vertebrae[mesh] OR Intervertebral Disk[mesh:noexp] OR (neck[tiab] OR necks[tiab] OR C1[tiab] OR C2[tiab] OR C3[tiab] OR C4[tiab] OR C5[tiab] OR C6[tiab] OR C7[tiab] OR T1[tiab] OR T2[tiab] OR T3[tiab] OR T4[tiab] OR T5[tiab] OR T6[tiab] OR T7[tiab] OR T8[tiab] OR T9[tiab] OR T10[tiab] OR T11[tiab] OR T12[tiab] OR odontoid[tiab] OR odontoids[tiab] OR occipital[tiab] OR occipitalis[tiab] OR occipitalisation[tiab] OR occipitalization[tiab] OR occipitalized[tiab] OR occipitalised[tiab] OR occipito-cervical[tiab] OR occipito-temporal[tiab] OR atlanto[tiab] OR atlanto-cervical[tiab] OR atlanto-occipital[tiab] OR atlanto-axial[tiab] OR atlanto-dental[tiab] OR brachial[tiab] OR cervico-brachial[tiab] OR cervico[tiab] OR “thoracic vertebr*”[tiab] OR “cervical vertebr*”[tiab] OR “thoracic spine*”[tiab] OR “cervical spine*”[tiab] OR “thoracic spinal”[tiab] OR “cervical spinal”[tiab] OR “thoracic outlet*”[tiab] OR “cervical outlet*”[tiab] OR “thoracic lumb*”[tiab] OR “cervical lumb*”[tiab] OR “thoracic disk*”[tiab] OR “cervical disk*”[tiab] OR “thoracic disc*”[tiab] OR “cervical disc*”[tiab] OR trapezius[tiab] OR cervicogenic[tiab] OR cervico-facial[tiab] OR cervico-thoracic[tiab] OR cervico-vertebral[tiab] OR cervico-spinal[tiab] OR cervical-brachial[tiab] OR cranio-vertebral[tiab]) | 864,534 |
| 3.  exclude uterus terms | genital diseases, female[mesh] OR Uterus[mesh] OR (genital disease*[tiab] OR uterus[tiab] OR uterine[tiab]) | 537,127 |
| 4. | #2 NOT #3 | 856,238 |
| 5.  Pain or injuries with pain as a symptom | spinal diseases[mesh] OR headache[mesh] OR pain[mesh] OR injuries[mesh] OR "Sprains and Strains"[mesh] OR Radiculopathy[mesh:noexp] OR Polyradiculopathy[mesh:noexp] OR Neuritis[mesh] OR temporomandibular joint disorders[mesh] OR temporomandibular joint dysfunction syndrome[mesh] OR Arthritis[mesh] OR Fibromyalgia[mesh:noexp] OR spondylitis[mesh:noexp] OR discitis[mesh:noexp] OR spondylosis[mesh:noexp] OR spondylolysis[mesh:noexp] OR spondylolisthesis[mesh:noexp] OR intervertebral disk degeneration[mesh:noexp] OR intervertebral disk displacement[mesh:noexp] OR (headache[tiab] OR headaches[tiab] OR pain[tiab] OR pains[tiab] OR pained[tiab] OR painful*[tiab] OR ache[tiab] OR aches[tiab] OR ached[tiab] OR aching[tiab] OR achy[tiab] OR achiness sore[tiab] OR soreness[tiab] OR stiff[tiab] OR stiffness[tiab] OR discomfort[tiab] OR discomforts[tiab] OR uncomfortable*[tiab] OR injury[tiab] OR injuries[tiab] OR injure[tiab] OR injured[tiab] OR injuring[tiab] OR sprain[tiab] OR sprains[tiab] OR sprained[tiab] OR strain[tiab] OR strains[tiab] OR strained[tiab] OR monoradiculopathy[tiab] OR monoradiculopathies[tiab] OR mononeuropathy[tiab] OR mononeuropathies[tiab] OR radiculopathy[tiab] OR radiculopathies[tiab] OR polyradiculopathy[tiab] OR polyradiculopathies[tiab] OR neuritis[tiab] OR neuropathy[tiab] OR neuropathies[tiab] OR neuropathic[tiab] OR radiculitis[tiab] OR “temporomandibular disorder*”[tiab] OR arthriti*[tiab] OR osteoarthriti*[tiab] OR fibromyalgia*[tiab] OR spondylosis[tiab] OR spondylitis[tiab] OR spondylolisthesis[tiab] OR spondylosis[tiab] OR spondylolysis[tiab] OR spondylolyses[tiab] OR spondylitis[tiab] OR spondylolisthesis[tiab] OR herniation*[tiab] OR herniate[tiab] OR herniates[tiab] OR herniated[tiab] OR herniating[tiab] OR slipped[tiab] OR prolapse[tiab] OR prolapses[tiab] OR prolapsed[tiab] OR prolapsing[tiab] OR displace[tiab] OR displaces[tiab] OR displaced[tiab] OR displacement*[tiab] OR bulg*[tiab] OR osteophytosis[tiab] OR discitis[tiab] OR diskitis[tiab] OR discopathy[tiab] OR discopathies[tiab] OR displacement*[tiab] OR degeneration*[tiab] OR degenerated[tiab] OR degenerating[tiab] OR neuralgia[tiab] OR neuralgias[tiab]) | 3,946,780 |
| 6. | #4 AND #5 | 236,645 |
| 7. | #1 OR #6 | 250,997 |
| 8.  diagnosis | "diagnosis" [Subheading]OR diagnosis[mesh] OR "Sensitivity and Specificity"[mesh] OR "Predictive Value of Tests"[mesh] OR (sensitiv*[tiab] OR specificit*[tiab] OR diagnos*[tiab] OR detect*[tiab] OR screening[tiab] OR screen[tiab] OR screened[tiab] OR "predictive value"[tiab] OR "predictive values") | 13,556,143 |
| 9. | #7 AND #8 | 164,504 |
| 10.  SR & meta-analysis filter | ("systematic review"[Publication Type] OR "meta analysis"[Publication Type] OR "systematic reviews as topic"[MeSH Terms] OR "Network Meta-Analysis"[Mesh] OR "meta analysis as topic"[MeSH Terms]) OR (“systematic review”[tiab] OR “meta-analysis”[tiab] OR “quantitative review”[tiab] OR “quantitative synthesis”[tiab] OR “pooled analysis”[tiab] OR “pooled analyses”[tiab] OR “meta-analysis”[tiab] OR “meta-analyses”[tiab] OR metanalysis[tiab] OR meta-regression*[tiab]) | 382,087 |
| 11. | #9 AND #10 | 2,453 |
| 12. | #11 AND ("2016"[Date - Publication] : "3000"[Date - Publication]) AND Journal Category: MEDLINE | 1,275 |

Web of Science (Clarivate), with the following selected databases:

Science Citation Index Expanded: 1900-present

Social Sciences Citation Index

Emerging Sources Index

Current chemical Reactions

Index Chemicus

Run 1/14/2022

| 1.  Neck pain | TS=(neckache OR neckaches OR neckpain OR neckpains OR whiplash OR whiplashes OR whiplashing OR whiplashed OR cervicodynia? OR cervicalgia? OR brachialgia? OR torticollis OR "myofascial pain syndrome" OR "myofascial pain syndromes" OR "thoracic outlet syndrome" OR "thoracic outlet syndromes" OR "thorax outlet syndrome" OR "spinal osteophytosis") | 11,868 |
| --- | --- | --- |
| 2.  Neck | TS=(neck OR necks OR C1 OR C2 OR C3 OR C4 OR C5 OR C6 OR C7 OR T1 OR T2 OR T3 OR T4 OR T5 OR T6 OR T7 OR T8 OR T9 OR T10 OR T11 OR T12 OR odontoid OR odontoids OR occipital OR occipitalis OR occipitali?ation OR occipitali?ed OR occipito?cervical OR occipito?temporal OR atlanto OR atlanto?cervical OR atlanto?occipital OR atlanto?axial OR atlanto?dental OR brachial OR cervico?brachial OR cervico OR ((thoracic OR cervical) near/3 (vertebra OR vertebrae OR vertebraes OR vertebral OR spine OR spines OR spinal OR outlet OR outlets OR lumbar OR lumbral OR disk OR disks OR disc OR discs)) OR trapezius OR cervicogenic OR cervico?facial OR cervico?thoracic OR cervico?vertebral OR cervico?spinal OR cervical?brachial OR cranio?vertebral) | 821,814 |
| 3. | TS=("genital disease*" OR uterus OR uterine) | 166,107 |
| 4. | #2 NOT #3 | 818,588 |
| 5. | TS=(headache OR headaches OR pain OR pains OR pained OR painful* OR ache OR aches OR ached OR aching OR achy OR achiness sore OR soreness OR stiff OR stiffness OR discomfort OR discomforts OR uncomfortable* OR injury OR injuries OR injure OR injured OR injuring OR sprain OR sprains OR sprained OR strain OR strains OR strained OR monoradiculopathy OR monoradiculopathies OR mononeuropathy OR mononeuropathies OR radiculopathy OR radiculopathies OR polyradiculopathy OR polyradiculopathies OR neuritis OR neuropathy OR neuropathies OR neuropathic OR radiculitis OR (temporomandibular NEAR/2 disorder?) OR arthriti? OR osteoarthriti? OR fibromyalgia? OR spondylosis OR spondylitis OR spondylolisthesis OR spondylosis OR spondylolysis OR spondylolyses OR spondylitis OR spondylolisthesis OR herniation? OR herniate OR herniates OR herniated OR herniating OR slipped OR prolapse OR prolapses OR prolapsed OR prolapsing OR displace OR displaces OR displaced OR displacement? OR bulg* OR osteophytosis OR discitis OR diskitis OR discopathy OR discopathies OR displacement? OR degeneration? OR degenerated OR degenerating OR neuralgia OR neuralgias) | 4,376,227 |
| 6. | #4 AND #5 | 163,528 |
| 7. | #1 OR #6 | 170,936 |
| 8. | TS=(sensitiv* OR specificit* or diagnos* OR detect* OR screening? OR screen? OR screened OR "predictive value" OR "predictive values") | 9,423,539 |
| 9. | #7 AND #8 | 51,760 |
| 10. | TS=("met analy*" OR metanaly* OR "meta regression*" OR metaregression* OR meta-analy* OR metaanaly* OR "systematic review*" OR cochrane) OR TS=(systematic* near/3 review*) OR TS=(quantitative near/3 (review* OR synthes*)) | 641,013 |
| 11. | #9 AND #10 | 1,621 |
| 12. | date filter: 2016 – present | 1,015 |

CINAHL Plus (EbscoHost)

| 1. | MH "Neck Pain" OR MH "Brachial Plexus Neuropathies+" OR MH "Neck Injuries+" OR MH "Whiplash Injuries" OR MH "Thoracic Outlet Syndrome" OR MH "Torticollis" OR MH "Brachial Plexus Neuritis" OR MH "Myofascial Pain Syndromes+" OR MH "Osteoarthritis, Spine+" OR MH "Spinal Osteophytosis" OR MH "Osteoarthritis, Cervical" OR TI(neckache OR neckaches OR neckpain OR neckpains OR whiplash OR whiplashes OR whiplashing OR whiplashed OR cervicodynia# OR cervicalgia# OR brachialgia# OR torticollis OR "myofascial pain syndrome" OR "myofascial pain syndromes" OR "thoracic outlet syndrome" OR "thoracic outlet syndromes" OR "thorax outlet syndrome" OR "spinal osteophytosis) OR AB(neckache OR neckaches OR neckpain OR neckpains OR whiplash OR whiplashes OR whiplashing OR whiplashed OR cervicodynia# OR cervicalgia# OR brachialgia# OR torticollis OR "myofascial pain syndrome" OR "myofascial pain syndromes" OR "thoracic outlet syndrome" OR "thoracic outlet syndromes" OR "thorax outlet syndrome" OR "spinal osteophytosis) | 16,762 |
| --- | --- | --- |
| 2. | MH "Neck+" OR MH "Neck Muscles+" OR MH "Cervical Plexus+" OR MH "Atlanto-Axial Joint" OR MH "Atlanto-Occipital Joint" OR MH "Cervical Atlas" OR MH "Spinal Nerve Roots+" OR MH "Brachial Plexus+" OR MH "Cervical Vertebrae+" OR MH "Thoracic Vertebrae" OR MH "Cervical Vertebrae+" OR MH "Intervertebral Disk+" OR TI(neck OR necks OR C1 OR C2 OR C3 OR C4 OR C5 OR C6 OR C7 OR T1 OR T2 OR T3 OR T4 OR T5 OR T6 OR T7 OR T8 OR T9 OR T10 OR T11 OR T12 OR odontoid OR odontoids OR occipital OR occipitalis OR occipitali#ation OR occipitali#ed OR occipito#cervical OR occipito#temporal OR atlanto OR atlanto#cervical OR atlanto#occipital OR atlanto#axial OR atlanto#dental OR brachial OR cervico#brachial OR cervico OR ((thoracic OR cervical) N3 (vertebra OR vertebrae OR vertebraes OR vertebral OR spine OR spines OR spinal OR outlet OR outlets OR lumbar OR lumbral OR disk OR disks OR disc OR discs)) OR trapezius OR cervicogenic OR cervico#facial OR cervico#thoracic OR cervico#vertebral OR cervico#spinal OR cervical#brachial OR cranio#vertebral) OR AB(neck OR necks OR C1 OR C2 OR C3 OR C4 OR C5 OR C6 OR C7 OR T1 OR T2 OR T3 OR T4 OR T5 OR T6 OR T7 OR T8 OR T9 OR T10 OR T11 OR T12 OR odontoid OR odontoids OR occipital OR occipitalis OR occipitali#ation OR occipitali#ed OR occipito#cervical OR occipito#temporal OR atlanto OR atlanto#cervical OR atlanto#occipital OR atlanto#axial OR atlanto#dental OR brachial OR cervico#brachial OR cervico OR ((thoracic OR cervical) N3 (vertebra OR vertebrae OR vertebraes OR vertebral OR spine OR spines OR spinal OR outlet OR outlets OR lumbar OR lumbral OR disk OR disks OR disc OR discs)) OR trapezius OR cervicogenic OR cervico#facial OR cervico#thoracic OR cervico#vertebral OR cervico#spinal OR cervical#brachial OR cranio#vertebral) | 137,579 |
| 3. | MH "Genital Diseases, Female+" OR MH "Uterus+" OR TI(genital disease* OR uterus OR uterine) OR AB(genital disease* OR uterus OR uterine) | 134,140 |
| 4. | S2 NOT S3 | 136,342 |
| 5. | MH "Spinal Diseases+" OR MH "Headache+" OR MH "Pain+" OR MH "Wounds and Injuries+" OR MH "Sprains and Strains+" OR MH "Radiculopathy" OR MH "Polyradiculopathy+" OR MH "Neuritis+" OR MH "Temporomandibular Joint Diseases+" OR MH "Temporomandibular Joint Syndrome" OR MH "Arthritis+" OR MH "Fibromyalgia" OR MH "Spondylosis+" OR MH "Discitis" OR MH "Spondylolisthesis" OR MH "Intervertebral Disk Displacement" OR TI (headache OR headaches OR pain OR pains OR pained OR painful* OR ache OR aches OR ached OR aching OR achy OR achiness sore OR soreness OR stiff OR stiffness OR discomfort OR discomforts OR uncomfortable* OR injury OR injuries OR injure OR injured OR injuring OR sprain OR sprains OR sprained OR strain OR strains OR strained OR monoradiculopathy OR monoradiculopathies OR mononeuropathy OR mononeuropathies OR radiculopathy OR radiculopathies OR polyradiculopathy OR polyradiculopathies OR neuritis OR neuropathy OR neuropathies OR neuropathic OR radiculitis OR (temporomandibular N2 disorder#) OR arthriti# OR osteoarthriti# OR fibromyalgia# OR spondylosis OR spondylitis OR spondylolisthesis OR spondylosis OR spondylolysis OR spondylolyses OR spondylitis OR spondylolisthesis OR herniation# OR herniate OR herniates OR herniated OR herniating OR slipped OR prolapse OR prolapses OR prolapsed OR prolapsing OR displace OR displaces OR displaced OR displacement# OR bulg* OR osteophytosis OR discitis OR diskitis OR discopathy OR discopathies OR displacement# OR degeneration# OR degenerated OR degenerating OR neuralgia OR neuralgias) OR AB(headache OR headaches OR pain OR pains OR pained OR painful* OR ache OR aches OR ached OR aching OR achy OR achiness sore OR soreness OR stiff OR stiffness OR discomfort OR discomforts OR uncomfortable* OR injury OR injuries OR injure OR injured OR injuring OR sprain OR sprains OR sprained OR strain OR strains OR strained OR monoradiculopathy OR monoradiculopathies OR mononeuropathy OR mononeuropathies OR radiculopathy OR radiculopathies OR polyradiculopathy OR polyradiculopathies OR neuritis OR neuropathy OR neuropathies OR neuropathic OR radiculitis OR (temporomandibular N2 disorder#) OR arthriti# OR osteoarthriti# OR fibromyalgia# OR spondylosis OR spondylitis OR spondylolisthesis OR spondylosis OR spondylolysis OR spondylolyses OR spondylitis OR spondylolisthesis OR herniation# OR herniate OR herniates OR herniated OR herniating OR slipped OR prolapse OR prolapses OR prolapsed OR prolapsing OR displace OR displaces OR displaced OR displacement# OR bulg* OR osteophytosis OR discitis OR diskitis OR discopathy OR discopathies OR displacement# OR degeneration# OR degenerated OR degenerating OR neuralgia OR neuralgias) | 983,088 |
| 6. | S4 AND S5 | 61,022 |
| 7. | S1 OR S6 | 68,349 |
| 8. | MW "DI" OR MH "Diagnosis+" OR MH "Sensitivity and Specificity" OR MH "Predictive Value of Tests" OR TI (sensitiv* OR specificit* or diagnos* OR detect* OR screening# OR screen# OR screened OR "predictive value" OR "predictive values") OR AB (sensitiv* OR specificit* or diagnos* OR detect* OR screening# OR screen# OR screened OR "predictive value" OR "predictive values") | 2,689,691 |
| 9. | S7 AND S8 | 46,671 |
| 10. | PT("Meta Analysis" OR "Meta Synthesis" OR "Systematic Review") OR MH "Meta Analysis" OR MH "Systematic Review" OR TI((systematic* N3 (review* OR overview*)) OR TI((methodologic* N3 (review* OR overview*)) OR TI((quantitative N3 (review* OR synthes*)) OR TI(met analy* OR metanaly* OR meta regression* OR metaregression* OR meta-analy* OR metaanaly* OR systematic review* OR cochrane)) OR AB((systematic* N3 (review* OR overview*)) OR AB((methodologic* N3 (review* OR overview*)) OR AB((quantitative N3 (review* OR synthes*)) OR AB(met analy* OR metanaly* OR meta regression* OR metaregression* OR meta-analy* OR metaanaly* OR systematic review* OR cochrane)) | 226,423 |
| 11. | S9 AND S10 | 1,603 |
| 12. | S11 AND Date filter: 2016 - present | 809 |
