# Supplementary material for: An overview of systematic reviews investigating clinical features for diagnosing neck pain and its associated disorders
Source: J Man Manip Ther. 2024 Dec 13;33(4):286–98. doi: 10.1080/10669817.2024.2436403 (PMC12281667; doi:10.1080/10669817.2024.2436403)
Supplement: Supplemental Appendix D_ROBIS Table.docx [file YJMT_A_2436403_SM6020.docx]

**Supplemental Appendix D**

| **Study Lead Author and Year** | **ROBIS Domain 1: Study Eligibility Criteria** | **ROBIS Domain 2: Identification and Selection of Studies** | **ROBIS Domain 3: Data Collection and Study Appraisal** | **ROBIS Domain 4: Synthesis and Findings** | **ROBIS Risk of Bias in the Review** |
| --- | --- | --- | --- | --- | --- |
| **Moser 2018^25^** | Low | Low | Low | Low | Low |
| **Malhotra 2017^53^** | Low | Low | Low | Low | Low |
| **Mansfield 2020^52^** | Low | Low | Low | Low | Low |
| **Gold 2017^41^** | Low | Low | High | Low | Low |
| **Mizer 2017^32^** | Low | Low | Low | Low | Low |
| **Paykin 2018^48^** | Unclear | Low | Low | Low | Low |
| **Lemeunier 2020^19^** | Low | Low | Low | Low | Low |
| **Lemeunier 2017^18^** | Low | Low | Low | Low | Low |
| **Lemeunier 2018^20^** | Low | Low | Low | Low | Low |
| **Liao 2020^54^** | High | Low | High | High | Low |
| **Miranda 2019^33^** | Low | Low | Low | Low | Low |
| **De Pauw 2016^38^** | Low | Low | Low | Low | Low |
| **Hill 2018^36^** | Low | Low | Low | Low | Low |
| **Moghaddas 2019^42^** | Low | Low | Low | Unclear | Low |
| **Usunier 2018^16^** | Low | Low | Low | Low | Low |
| **Farrell 2019^14^** | Low | Low | Low | Low | Low |
| **Yang 2020^37^** | Low | Low | Low | Low | Low |
| **Manchikanti 2018^43^** | High | High | High | Unclear | Unclear |
| **Abichandani 2023^35^** | Low | Low | Low | Low | Low |
| **Varga 2023^44^** | Low | Low | High | Low | High |
| **Vazirizadeh-Mahabadi 2023^49^** | Low | Low | Low | Low | Low |
| **Romeo 2022^33^** | Low | Low | Low | Low | Low |
| **Peng 2022^39^** | Low | Low | Low | Low | Low |
| **Lindenmann 2022^45^** | High | High | High | Unclear | Unclear |
| **Franov 2022^40^** | Low | Low | Low | Low | Low |
| **Owers 2017^47^** | Low | Low | Low | Low | Low |
