# Supplementary material for: An overview of systematic reviews investigating clinical features for diagnosing neck pain and its associated disorders
Source: J Man Manip Ther. 2024 Dec 13;33(4):286–98. doi: 10.1080/10669817.2024.2436403 (PMC12281667; doi:10.1080/10669817.2024.2436403)
Supplement: Supplemental Appendix B_Neck Pain Clinical Features Associations and Risk Factors.docx [file YJMT_A_2436403_SM6019.docx]

**SUPPLEMENTAL APPENDIX B**

| **Nonspecific/Mechanical Neck Pain** | | |
| --- | --- | --- |
| **Test/Measure** | **Studies (author last name and year)** | ***Relevant Statistics Reported*** |
| Posture | Lemeunier 2018^20^ | ***Validity for Head Posture (MD; NAD vs Controls)***  *Visual Assessment - Photographic Method*  *Craniovertebral (degrees)*  -0.60 (95% CI; -3.77, 2.57)  *High Thoracic (degrees)*  1.30 (95% CI;-1.96, 4.56)  *Shoulder protrusion (mm)*  0.60, p = 0.85  *Visual Assessment - Caliper OR Inclinometer*  -1.30 (cm) (95% CI; -3.13, 0.53)  *Visual Assessment - Goniometer*  *Head Position (degrees)*  4.40 (95% CI; 1.99, 6.81)  *Craniovertebral (CV) angle*  -5.09 (95% CI; -6.91, -3.27)  ***Association between CV angle and NPQ***  Pearson’s r = -0.31, p = 0.015  ***Association between CV angle and NPRS***  Pearson’s r = -0.19 ; p = 0.54 |
| Cervical joint referral patterns, or ‘‘pain maps” | Usunier 2018^16^ | **Segment Prediction - Confirmed with Joint Block**  83%  **Prevalence of Cervical Joint Referral**  36% (95% CI: 27-45%) |
| Neck Functional Tests | Lemeunier 2020^19^ | ***Construct Validity MD Neck Pain vs. Controls***  *Active shoulder control tests in elderly computer workers*  *Shoulder elevation, right (N):*  -44.00 (95% CI; 51.38, 36.62)  *Shoulder elevation, left (N)*:  -66.00 (95% CI: 77.46, 54.54)  *Handgrip strength in elderly computer workers*  *Right (N):*  17.00 (95% CI: 21.35, 12.65)  *Left (N):*  6.00 (95% CI: 12.61, 0.61)  ***Association between lifting overhead (kg) and reference measures***  *Pain:*  r = 0.39 (95% CI: 0.48, 0.29)  *SFS:*  r = 0.60 (95% CI: 0.52, 0.66)  *NDI:*  r = 0.39 (95% CI: 0.48, 0.29)  *HADS A:*  r = 0.27 (95% CI: 0.37, 0.16)  *HADS D:*  r = 0.30 (95% CI: 0.40, 0.20)  ***Association between***  ***overhead working (seconds) and***  ***reference measures***  *Pain*:  r = 0.36 (95% CI: 0.46, 0.26)  *SFS:*  r = 0.61 (95% CI: 0.54, 0.68)  *NDI:*  r = 0.45 (95% CI: 0.53, 0.35)  *HADS A:*  r = 0.36 (95% CI: 0.45, 0.26)  ***Association between right***  ***hand grip strength (kg F) and***  ***reference measures***  *Pain*  r = -0.26 (95% CI; 0.36, 0.16)  *Functional Ability (SFS)*  r = 0.38 (95% CI; 0.28, 0.47)  *Disability (NDI)*  r = -0.26 (95% CI; 0.36, 0.15)  *Anxiety (HADS-A)*  r = - 0.28 (95% CI: 0.38, 0.17)  *Depression (HADS-D)*  r = - 0.25 (95% CI: 0.35, 0.15) |
| Cervical Strength | Lemeunier 2020^19^ | **Neck pain vs healthy controls**  *Cranio-Cervical Flexion Test (CCFT)*  *Pain*  r = -0.29 (p >0.05), -0.37 (p = 0.02)  *Disability (NDI)*  r = -0.24 (p>0.05), -0.40 (p<0.01)  *Cervical Muscle Strength*  *MD in muscle strength (kg):*  *Flexion:* 3.39 (95% CI: 1.76-5.03)  Cohen’s d = 0.965; p<0.05  *Extension*: 4.82 (95% CI: 2.93–6.71)  Cohen’s d = 1.176; p< 0.05  *Lateral flexion*: 3.25 (95% CI: 1.75–4.76);  Cohen’s d: 1.046; p< 0.05 |
|  | Romeo 2022^34^ | **Non-specific Neck Pain vs Asymptomatic**  Cranio-Cervical Flexion Test (mmHG)  **Muscle Activation in Asymptomatic Subjects - Deep Cervical Flexors**  *Pooled EMG Activity*  22 mmHG: 32.66 (95% CI; 22.44, 44.85)  24 mmHG: 42.58 (95% CI; 34.81,50.72)  26 mmHG: 52.55 (95% CI; 45.73,59.28)  28 mmHG: 62.07 (95% CI; 54.65,68.96)  30 mmHG: 77.69 (95% CI; 64.41,87.01)  *Pooled US - Muscle Thickness*  22 mmHG: 3.99 (95% CI; 2.15,7.28)  24 mmHG: 10 (95% CI; 6.55,14.97)  26 mmHG: 14.75 (95% CI; 10.2,20.87)  28 mmHG: 19.01 (95% CI; 14.77,24.12)  30 mmHG: 21.47 (95% CI; 16.14,27.98)  *Pooled EMG and US*  22 mmHG: 17.24 (95% CI; 4.83,46.08)  24 mmHG: 27.42 (95% CI; 10.19,55.73)  26 mmHG: 30.73 (95% CI;13.05,56.73)  28 mmHG: 38.54 (95% CI;15.88,67.56)  30 mmHG :43.48 (95% CI;13.08,79.72)  **Muscle Activation in Nonspecific Neck Pain - Deep Cervical Flexors**  *Pooled EMG and US*  22 mmHG: 8.4 (95% CI; 1.2,40.88)  24 mmHG: 12.11 (95% CI; 1.47,55.89)  26 mmHG: 14.57 (95% CI; 2.49,53.24)  28 mmHG: 16.8 (95% CI; 3,56.84)  30 mmHG: 21.86 (95% CI; 3.61,67.64)  **Muscle Activation in Asymptomatic Subjects - SCM**  *Pooled EMG Activity*  22 mmHG: 7.44 (95% CI; 5.55,9.9)  24 mmHG: 11.63 (95% CI; 8.15,16.33)  26 mmHG: 17.12 (95% CI; 11.11,25.45)  28 mmHG: 24.5 (95% CI; 14.61,38.08)  30 mmHG: 33.91 (95% CI; 23.2,46.56)  *Pooled US - Muscle Thickness*  22 mmHG: 4 (95% CI; 1.45,10.58)  24 mmHG: 9.82 (95% CI; 3.44,24.94)  26 mmHG: 18.81 (95% CI; 10.85,30.59)  28 mmHG: 22.42 (95% CI; 11.55,39)  30 mmHG: 28.85 (95% CI; 15.01,48.21)  *Pooled EMG and US*  22 mmHG: 7.16 (95% CI; 5.41,9.44)  24 mmHG: 11.46 (95% CI; 8.19,15.82)  26 mmHG: 17.33 (95% CI; 11.92,24.5)  28 mmHG: 24 (95% CI; 15.47,35.26)  30 mmHG: 32.71 (95% CI; 23.54,43.42)  **Muscle Activation in Nonspecific Neck Pain Subjects - SCM**  *Pooled EMG Activity*  22 mmHG: 12.17 (95% CI; 6.46,21.76)  24 mmHG: 22.66 (95% CI; 13.99,34.55)  26 mmHG: 32.98 (95% CI; 24.82,42.31)  28 mmHG: 46.1 (95% CI; 31.48,61.42)  30 mmHG: 55.55 (95% CI; 37.52,72.24)  *Pooled EMG and US*  22 mmHG: 11.35 (95% CI; 6.46,19.21)  24 mmHG: 21.62 (95% CI; 14.59,30.81)  26 mmHG: 32.05 (95% CI; 25.24,39.72)  28 mmHG: 43.31 (95% CI; 31.29,56.17)  30 mmHG: 53.42 (95% CI; 38.9,67.38)  **Muscle Activation in Asymptomatic Subjects - Anterior Scalene**  *Pooled EMG Activity*  22 mmHG: 15.63 (95% CI; 12.53,19.32)  24 mmHG: 17.99 (95% CI; 12.39,25.38)  26 mmHG: 22.73 (95% CI; 14.6,33.61)  28 mmHG: 33.27 (95% CI; 17.2,54.47)  30 mmHG: 33.8 (95% CI; 14.95,59.74)  **Muscle Activation in Nonspecific Neck Pain Subjects - Anterior Scalene**  *Pooled EMG Activity*  22 mmHG: 11.27 (95% CI; 8.84,14.28)  24 mmHG: 23.09 (95% CI; 19.02,27.72)  26 mmHG: 35.62 (95% CI; 27.79,44.3)  28 mmHG: 53.66 (95% CI; 38.81,67.88)  30 mmHG: 67.99 (95% CI; 50.98,81.27)  **Variation in Craniocervical Flexion Motion by Pressure Level**  *Asymptomatic - Pooled*  22 mmHG: 29.2 (95% CI; 21.6,38.2)  24 mmHG: 47.7 (95% CI; 35.1,60.6)  26 mmHG: 62 (95% CI; 49.3,73.3)  28 mmHG: 73.2 (95% CI; 61,82.7)  30 mmHG: 84.6 (95% CI; 67.6,93.5)  *Nonspecific Neck Pain*  22 mmHG: 19.9 (95% CI; 17.5,22.3)  24 mmHG: 35.6 (95% CI; 32.4,38.8)  26 mmHG: 50.3 (95% CI; 46.7,53.9)  28 mmHG: 62.8 (95% CI; 59.2,66.4)  30 mmHG: 70.9 (95% CI; 67.8,74.0)  **Correlation with Disability (NDI/NPNPQ)**  Pooled CCFT1: -0.26 (95% CI; -0.40, -0.00); I2 = 0%  Pooled CCFT1 and AS: -0.21 (95% CI: -.043, 0.06); I2 = 0%  Pooled CCFT PI and 2: -0.20 (-0.43, 0.06) I2 = 0%  **Correlation with Pain (VAS)**  Pooled CCFT 1: -0.24 ( 95% CI; -0.47, 0.01); I2 = 0%  Pooled CCFT AS and 1: -0.14 ( -0.38, 0.11); I2 = 30.2%  POOLED CCFT2: -0.15 (-0.48, 0.21); I2 = 50.3%  Pooled CCFT PI and 2: -0.08 ( 95% CI; -0.27, 0.11); I2 = 22.6%  **Trends in Variation of Muscle Activity for 2 mmHG increases**  *Asymptomatic - SCM*  EMG: 6.58 (95% CI; 4.66–8.5)  US: 6.23 (95% CI; 4.9–7.56)  EMG + US: 6.36 (95% CI; 4.86–7.87)  *Nonspecific Neck Pain - SCM*  EMG: 11.02 (95% CI; 10.09–11.95)  US: 9.1 (95% CI; 7.16–11.04)  EMG + US: 10.58 (95% CI; 10.27–10.9)    *Asymptomatic - Anterior Scalene*  EMG: 5.16 (95% CI; 2.5–7.82)  *Nonspecific Neck Pain - Anterior Scalene*  EMG: 14.4 (95% CI; 12.33–16.47)  *Asymptomatic - Deep Cervical Flexor*  EMG: 10.96 (95% CI; 8.87–13.04)  US: 4.4 (95% CI; 3.21–5.59)  EMG + US: 6.36 (95% CI; 4.6–8.12)  *Nonspecific Neck Pain - Deep Cervical Flexor*  EMG: 5.29 (95% CI; 2.74–7.84)  US: 1.5 (95% CI; 1.18–1.82)  EMG + US: 3.16 (95% CI; 2.31–4.01) |
|  | Abichandani 2023^35^ | ***Standard Error of Measure (N)***  *Isometric Dynamometer*  *Cranio-Cervical Flexors (MVIC peak torque)*  Inner: 0.7  Middle: 1  Outer: 1  *Hand-Held Dynamometer*  Sternocleidomastoid  Left: 1.82  Right: 1.67  ***Minimal Detectable Change (N)***  *Hand-Held Dynamometer*  *Sternocleidomastoid*  Left: 3.16  Right: 3.56  Flexion: 8.7  Extension: 12.5  Left side flexion: 6.3  Right side flexion: 7.2  **Coefficient of Variance (%)**  *Strain-Gauge Dynamometer*  Neck Flexors : 6.5 (p>0.05)  Neck Extensors: 13 (p>0.05) |
|  | Miranda 2019^33^ | **Chronic Neck Pain vs. Controls (Newtons)**  *Flexion*  SMD = -0.90 (95% CI; -1.13 to -0.67)  *Extension*  SMD = -0.79 (95% CI; -0.99 to -0.60)  *Right Lateral Flexion*  SMD = -0.74 (95% CI; -1.03 to -0.45)  *Left Lateral Flexion*  SMD = -0.75 (95% CI; -1.04 to -0.46) |
| Cervical Endurance | Lemeunier 2020^19^ | ***Validity (neck pain vs controls)***  *Chin tuck neck flexion test*  Median difference (seconds):  Neck pain = 18.82  Control = 26.29  *NET*  Median difference (minutes):  Neck pain = 3.44  Control = 3.54  *NME*  Prone NME and pain correlation  r = -0.30; p = 0.01  Supine NME and disability correlation  r = -0.23; p = 0.07  *DCE*  Median difference (seconds):  29.21; p = 0.06) |
| Magnetic Resonance Imaging (MRI) | Hill 2018^36^ | ***Predictor of Symptoms***  **Current Neck Pain**  ***Risk Estimate MD***  *Disc protrusion (grade 1-4 vs 0)*  *Pain at 1 year*  -1.83 (95% CI: -0.42, -3.23)  *Pain at 5 years*  -2.88(95% CI: -1.5, -4.26)  *Disc Protrusions (grade 2-4 vs 0-1)*  *Pain at 5 years*  -2.49 (95% CI: -0.97, -4.01)  *Disc Protrusions (grade 3-4 vs 0-2)*  *Pain at 1 year*  -1.84 (95% CI: -0.43, -3.26)  *Pain at 5 years*  -2.51 (95% CI: -0.98, -4.05)  ***Risk Estimate Risk Ratio***  *Mild Disc Degeneration*  *Pain at 1 year*  0.59 (95% CI: 0.36, 0.98)  *Moderate-Severe Disc Degeneration*  *Pain at 1 year*  0.46 (95% CI: 0.25 to 0.87)  *Muscle Fatty Infiltration*  *DIsability at 3 Months*  21.00 (95% CI: 2.97 to 148.31)  **No Neck Pain**  *Foraminal Stenosis*  Risk estimate risk ratio *- pain at 10 years*  2.99 (95% CI: 1.23 to 7.23) |
|  | Yang 2020^37^ | **Modic Change-Neck Pain Association**  OR = 5.356 (95% CI: 1.31-12.80); p<0.001  OR = 3.268 (95% CI: 1.26-8.51), p=0.015  **Modic Change/Disc Degeneration**  OR = 3.9 (95% CI: 2.42-6.3)  Extrusion RR = 2.42 (95% CI: 1.93-3.04)  **Modic Change/Neck Pain/Disc Degeneration**  Persistent Neck Pain  OR = 2.308 (95% CI: 1.244-4.282) p<0.05  Severe Disc Degeneration  OR = 2.423 (95% CI: 1.169-5.023) p<0.05  Neck Pain  OR = 2.71 (95% CI: 1.08-6.80) p=0.033  Posterior Disc Protrusions  OR = 3.31 (95% CI: 1.21-9.05) p=0.020 |
|  | Farrell 2019^14^ | **Chronic NSNP vs controls**  *CSA of C2-3 multifidus*  SMD = -0.30 (95% CI: -1.12, 0.51), p = 0.47  Average left and right C5-6 MD = -32.70 mm^2^ (95% CI: -48.81, -16.59)  *CSA of SCM*  C2-3 SMD = 0.78 (95% CI: -0.65, 2.21), p = 0.29  C5-6 SMD = 1.10 (95% CI: -1.29, 3.49), p = 0.37  Average left and right C2-3 MD = 47.60 cm^2^ (95% CI: 31.11, 64.09)  Average left and right C5-6 MD = 74.10 cm^2^ (95% CI: 57.68, 90.52)  *CSA of semispinalis capitis at C2-3*  SMD = -0.21 (95% CI: -1.88, 1.45), p = 0.80  Average left and right C2-3 MD = -32.50 mm^2^ (95% CI: -48.67, -16.33)  Average left and right C5-6 MD = -49.10 mm^2^ (95% CI: -65.28, -32.92)  *CSA of longus capitis/colli at C2-3*  SMD = 0.04 (95% CI: -0.39, 0.47), p = 0.87  *CSA of rectus capitis posterior major at C1-2*  SMD = -1.18 (95% CI: -1.65, -0.71), p < 0.001  *CSA of rectus capitis posterior minor at C1-2*  SMD = 0.54 (95% CI: -1.09, 0.01), p = 0.06  *CSA of splenius capitis*  C2-3: SMD = 0.05 (95% CI –1.59, 1.68), p = 0.96  C5-6: SMD = –0.92 (95% CI –3.04, 1.20), p = 0.40  Average left and right C2-3 MD = -23.70 mm^2^ (95% CI: 39.88, -7.52)  Average left and right C5-6 MD = -61.90 mm^2^ (95% CI: -78.01, -45.79)  *CSA - Semispinalis Cervicis and Multifidus (intermittent neck pain in previous year)*  Left C5-6 MD = 0.79 cm^2^(95% CI: 0.39, 1.19)  Right C5-6 MD = 0.55 cm^2^ (95% CI: 0.19, 0.91)  *CSA - Semispinalis Cervicis*  Average left and right C5-6 MD = -56.80 mm^2^ (95% CI: -72.97, -40.63)  *CSA - Scalenus Anterior*  Right C5-6 MD = 0.05 cm^2^ (95% CI: 0.01, 0.09)  *CSA - Rectus Capitis Posterior Major*  Average left and right MD = -34.60 mm^2^ (95% CI: -50.71, -18.49)  *CSA* - *Rectus Capitis Posterior Minor*  Average left and right MD = -23.70 mm^2^ (95% CI: -39.66, -7.74)  *CSA with MFI Subtracted - Multifidus*  Average left and right C5-6 MD = -22.80 mm^2^ (-35.93, -9.66)  *CSA with MFI Subtracted - Semispinalis Cervicis*  Average left and right C2-3 MD = 15.50 mm^2^ (95% CI: 2.43, 28.57)  Average left and right C5-6 MD = -40.50 mm^2^ (95%CI: -53.63, -27.37)  *CSA with MFI Subtracted - Semispinalis Capitis*  Average left and right C2-3 MD = -19.70 mm^2^ (95% CI: -32.83, -6.57)  Average left and right C5-6 MD = -35.20 mm^2^ (95% CI: -48.33, -22.07)  *CSA with MFI Subtracted - Splenius Capitis*  Average left and right C5-6 MD = -44.30 (95%CI: -57.36, -31.24)  *CSA with MFI Subtracted - SCM*  Average Left and Right C2-3 MD = 51.00 mm^2^ (95%CI: 37.75, 64.25)  Average Left and Right C5-6 MD = 73.10 mm^2^ (95%CI: 59.78, 86.42)  *CSA with MFI Subtracted - Rectus Capitis Posterior Major*  Average left and right MD = -25.40 mm^2^ (95% CI: -38.40, -12.40)  *CSA with MFI Subtracted - Rectus Capitis Posterior Minor*  Average left and right MD = -19.30 mm^2^ (95%CI: -32.36, -6.24)  *MFI - Rectus Capitis Posterior Major*  Average left and right MD = 0.05 (95%CI: 0.02, 0.08)  *MFI - Rectus Capitis Posterior Minor*  Average left and right MD = 0.04 (95%CI: 0.02, 0.06)  *Intervertebral Disc Degeneration (Pfirrman grading)*  Chronic neck pain MD = 1.20 (95%CI: 1.07, 1.33)  *Spinal Cord Compression (Hayashi Grading)*  MD = 0.50 (95% CI: 0.23, 0.77)  *Vertebral Body - Modic Changes*  OR = 2.00 (95% CI: 1.42, 2.82)  *Disc Degeneration*  OR = 0.84 (95% CI: 0.57, 1.24); p = 0.39  Modic Changes  OR = 0.92 (95% CI: 0.13, 6.62), p = 0.94 |
| Discography | Manchikanti 2018^43^ | **Clinical Prevalence of Pain Source in Chronic Neck Pain**  Discogenic: 16-53%  Zygapophyseal: 41-55% |
| Single-Photon Emission Computed Tomography Imaging (SPECT) | Varga 2023^44^ | ***SPECT vs MRI overall agreement***  *Increased Vertebral Body Uptake*  K = 0.571  *Facet Arthropathy*  K = 0.333  ***Prevalence of pain generator***  Facet Arthropathy: 52%  Degenerative Disc Disease: 36%  ***Improvement of pain with SPECT+ injections vs SPECT-***  *OR = 0.68 (95% CI: 0.36 to 1.26)* |
| Multiple Imaging Modalities | Gold 2017^41^ | **Biomarker Correlations of Longus Colli**  *NDI and Longus Colli CSA, dominant side*  Rho = −0.45, p = 0.05,  *NDI and Longus Colli CSA, non-dominant side*  Rho = −0.48, p = 0.03  *NDI and and Longus Colli APD, dominant side*:  rho = −0.49, p = 0.03,  *NDI and and Longus Colli APD, non-dominant side*  rho = −0.45, p = 0.05 |
|  | Peng 2022^39^ | ***Chronic Nonspecific Neck Pain vs. Controls (MD)***  *Longus Colli*  **CSA**: -0.23 (95%CI; -0.37, -0.08);  T2 = 0.01; Chi2 = 19.75; df = 2 (P< 0.0001); I2 = 90%; Z = 3.04 (P = 0.002)  **LD**: 0.47 (95% CI; -0.45, 1.38)  Chi2 = 1.52; df = 1 (P = 0.2)2; I2 = 34%; Z = 1 (P = 0.32)  APD: -0.88 (95% CI; -1.42, -0.33)  Chi2 = 0.86; df = 1 (P = 0.35); I2 = 0%; Z = 3.16 ( P = 0.002)  Shape Ratio: 0.18 (95% CI; 0.05, 0.32)  Chi2 = 0.86; df = 1 (P = 0.17); I2 = 46%; Z = 2.60 ( P = 0.009)  *Semispinatus Capitus*  LD: 0.03 (95% CI; -0.13, 0.18)  Chi2 = 0.98; df = 1 ( P = 0.32); I2 = 0%; Z = 0.35 ( P = 0.72)  APD: 0.02 (95% CI; -0.01, 0.05)  Chi2 = 0.98; df = 1 (P = 0.32); I2 = 0%; Z = 2.36 ( P = 0.02)  MLD: -0.19 (95% CI; -0.34, -0.03)  Chi2 = 0.98; df = 1 (P = 0.32); I2 = 0%; Z = 2.36 ( P = 0.02)  *Multifidus*  CSA: -1.06 (95% CI; -0.41, 0.10)  T2 = 0.03; Chi2 = 13.68; df = 1 (P = 0.0002); I2 = 93%; Z = 1.21 ( P = 0.23) |

Abbreviations: MD - Mean Difference; NAD - Neck Associated Disorders; CV - craniovertebral; NPQ - Neck Pain Questionnaire; NPNPQ - Northwick Park Neck Pain Questionnaire; NPRS - Numeric Pain Rating Scale; CROM - Cervical Range of Motion; AROM - Active Range of Motion; NDI - Neck Disability Index; PROM - Passive Range of Motion; OS-mPNFT - Onset of Symptom in the modified version of the Passive Neck Flexion Test; SP-mPNFT - Submaximal Pain in the modified version of the Passive Neck Flexion Test; N - Newtons; kg - Kilograms; SFS - Spinal Functional Sort; HADS A - HADS A: Hospital Anxiety and Depression Scale, subscale Anxiety; HADS D - Hospital Anxiety and Depression Scale, subscale Depression; EMG - Electromyography; US - Ultrasound; SCM - Sternocleidomastoid; MVIC - Maximal Voluntary Isometric Contraction; SEM - Standard Error of Measurement; Nm - Newtons meter; MDC - Minimal Detectable Change; SMD - Standardized Mean Difference; NET - Neck Extensor Test; NME - ;Neck Muscle Endurance Test; DCE - Deep Cervical Extensor TEst; OR - Odds Ratio ; RR - Risk Ratio; NSNP - Non-specific Neck Pain; CSA - Cross-sectional Area; MFI - Muscle Fat Infiltration; MRI - Magnetic Resonance Imaging

| **Whiplash Associated Disorders (WAD)** | | |
| --- | --- | --- |
| **Test/Measure** | **Studies (author last name and year)** | **Relevant Statistics Reported** |
| Morphological changes | Farrell 2019^14^ | ***Acute WAD***  *Intervertebral Disc Height Loss*  Males (≥40 years) OR = 0.47 (95% CI: 0.30, 0.73)  Total (≥40 years) OR = 0.56 (95% CI: 0.41, 0.77)  *Intervertebral Disc Protrusion (Anterior)*  Total (anterior protrusion) OR = 0.64 (95% CI: 0.49, 0.84)  Males (≥40 years) OR = 0.58 (95% CI: 0.40, 0.83)  *Intervertebral Disc Protrusion (Posterior)*  Total (posterior protrusion) OR = 1.20 (95% CI: 0.99, 1.46)  Females (≥40 years) OR = 1.44 (95% CI: 1.05, 1.97)  *Intervertebral Disc Signal Changes*  Males (≥40 years) OR = OR 1.55 (95% CI: 1.22, 1.96)  Females (≥40 years) OR = OR 0.67 (95% CI: 0.52, 0.87)  *Transverse Ligament Size - Sagittal Plane*  Males - MD = 0.19 mm^2^ (p = 0.03)  *Transverse Ligament Size - Transverse Plane*  Males - MD = 0.13mm^2^ (p = 0.04)  *CSA - Multifidus*  Combined Left and Right C3-4 MD = 56.10 mm^2^ (95% CI: 23.06, 89.14)  Combined Left and Right C4-5 MD = 46.40 mm^2^ (95% CI: 10.61, 82.19)  Combined Left and Right C5-6 MD = 49.00 mm^2^ ( (95% CI: 11.95, 86.05)  *CSA - Semispinalis Cervicis*  MD = 61.50 mm^2^ (95% CI: 11.95, 111.05)  CSA  *Multifidus*  MD = 46.40 (95% CI: 10.61, 82.19) mm^2^ to 56.10 (23.06, 89.14) mm  *Semispinalis Cervicis*  MD = 61.50 (95% CI: 11.95, 111.05) mm^2^  *Muscle Strain*  OR = 2.69 (95% CI: 1.16, 6.21)  *Vertebral Body Occult Fracture*  OR = 8.61 (95%: 1.06, 70.17)  **Chronic WAD**  *Facet Joint Synovial Fold Anterior-Posterior Length*  C1-2 mm dorsal: p <0.01  C1-2 % ventra:l p = 0.01  C1-2 % dorsal: p <0.01  *Facet Joint Synovial Fold Composition*  Facet joints dorsal: OR = 2.02 (95% CI: 1.17, 3.48)  *CSA - Multifidus*  Average left and right C5-6 MD: 22.70 mm^2^ (95% CI: 10.08, 35.32)  *CSA - Semispinalis Cervicis*  Average left and right C2-3 MD = -18.70 mm^2^ (95% CI: -31.32, -6.08)  Average left and right C5-6 MD = -22.50 mm^2^ (95% CI: -32.26, -9.74)  *CSA - Semispinalis Capitis*  Average left and right C2-3 MD = 40.10 mm^2^ (95% CI: 27.40, 52.80)  Average left and right C5-6 MD = -18.70 mm^2^ (95% CI: -31.32, -6.08)  *CSA - Splenius Capitis*  Average left and right C2-3 MD = 24.60 mm^2^ (95% CI: 11.92, 37.28)  *CSA - Upper Trapezius*  Left C3 MD = 11.80 mm^2^ (95% CI: 0.43, 23.17)  Right C7 MD = -198.80 mm^2^ (95% CI: -372.41, -25.19)  *CSA - Rectus Capitis Posterior Major*  Right MD = 16.40 mm^2^ (95% CI: 0.05, 32.75)  *CSA - Longus Capitis*  Average left and right C2-3 MD = 24.80 mm^2^ (95% CI: 11.41, 38.19)  Average left and right C5-6 MD = 23.50 mm^2^ (95% CI: 10.19, 36.81)  *CSA - SCM*  Average left and right C2-3 MD = 19.30 mm^2^ (95% CI: 6.23, 32.37)  Average left and right C5-6 MD = 29.40 mm^2^ (95% CI: 16.33, 42.47)  *CSA - Longus Capitis and SCM*  Average C0-1, C2-3, C5-6 MD = 23.10 mm^2^ (95% CI: 14.30, 31.90)  *CSA with MFI Subtracted - Semispinalis Cervcis*  Average left and right C2-3 MD = -12.60 mm^2^ (95% CI: -22.78, -2.43)  Average left and right C5-6 MD = -26.60 mm^2^ (95% CI: -36.85, -16.35)  CSA with MFI Subtracted - Semispinalis Capitis  Average left and right C5-6 MD = -21.40 mm^2^ (95% CI: -31.65, -11.15)  *CSA with MFI Subtracted - Splenius Capitis*  Average left and right C5-6 MD = -16.60 mm^2^ (95% CI: -26.85, -6.35)  *CSA with MFI Subtracted - SCM*  Average left and right C5-6 MD = 21.00 mm^2^ (95% CI: 10.45, 31.55)  *CSA with MFI Subtracted - Rectus Capitis Posterior Major*  Average left and right MD = -13.80 mm^2^ (95% CI: -23.98, -3.63)  *CSA with MFI Subtracted - Rectus Capitis Posterior Minor*  Average left and right MD = -18.90 mm^2^ (95% CI: -29.15, -8.65)  *MFI - Multifidus and Semispinalis Combined*  Average left and right C3 MD = 0.09 mm^2^ (95% CI: 0.01, 0.17)  Average left and right C5 MD = 0.08 mm^2^ (95% CI: 0.02, 0.14)  *MFI - Multifidus Only*  Average Left and Right C4 = MD 0.12 mm^2^ (95% CI: 0.10, 0.14)  Average Left and Right C5 = MD 0.10 mm^2^ (95% CI: 0.09, 0.11)  Average Left and Right C6 = MD 0.09 mm^2^ (95% CI: 0.07, 0.11)  Average Left and Right C7 = MD 0.12 mm^2^ (95% CI: 0.10, 0.14)  *MFI - Semispinalis Cervicis*  p <0.0001  *MFI - Semispinalis Capitis*  p <0.0001  *MFI - Splenius Capitis*  p <0.0001  *MFI - Upper Trapezius*  p <0.0001  *MFI - Rectus Capitis Posterior Major*  p <0.0001  *MFI - Rectus Capitis Posterior Minor*  p <0.0001  *MFI - Longus Capitis +/- Colli*  Average left and right C2-3 MD = 0.09 mm^2^ (95% CI: 0.07, 0.11)  Average left and right C5-6 MD = 0.09 mm^2^ (95% CI: 0.07, 0.11)  *MFI - SCM*  Average left and right C2-3 MD = 0.03 (95% CI: 0.01, 0.05)  *Oropharynx CSA*  MD = -69.70 mm^2^ (95% CI: -86.81, -52.59)  *Oropharynx Shape Ratio (anterior-posterior: lateral)*  MD = -0.27 mm^2^ (95% CI: -0.36, -0.18)  ***Chronic WAD vs Controls - Pooled CSA Changes***  *C4 Multifidus*  SMD = 0.54 (95% CI; –0.95, 2.04)  p =0.48  *C5 Multifidus*  SMD = 0.70 (95% CI; –0.40, 1.80)  p = 0.21  *C6 Multifidus*  SMD = 0.36 (95% CI; –0.08, 0.80)  p = 0.10 |
|  | Owers 2018^47^ | ***Chronic WAD***  *Multifidus CSA*  C5 (Z=3.51,p<0.01)  C6 (Z=2.66,p<0.01)  *Muscle Fatty Infiltrates* (MFI)  C7 (Z=2.52,p<0.01) (I2=83%). |
| Cervical Strength | Abichandani 2023^35^ | ***Standard Error of Measure (N)***  *Multi-Cervical Unit*  Flexion: 7.5  Extension: 6.2  Protraction: 13.7  Retraction: 14.6  Left lateral flexion: 6.2  Right lateral flexion: 6.4  Composite: 9.1 ± 3.9  ***Minimal Detectable Change (N)***  *Multi-Cervical Unit*  Flexion: 17.5  Extension: 14.5  Protraction: 31.9  Retraction: 33.9  Left lateral flexion: 14.4  Right lateral flexion: 14.8  Composite: 21.1 ± 9.2  **Coefficient of Variance (%)**  *Multi-Cervical Unit*  Flexion: 18.9  Extension: 11.2  Protraction: 30.9  Retraction: 29.9  Left lateral flexion: 16.2  Right lateral flexion: 18.9  Composite: 21 ± 7.8 |

Abbreviations: CSA - cross sectional area; MD - mean difference; SMD - standard mean difference; SCM - sternocleidomastoid; MFI - muscle fat infiltration; N - Newtons; CROM - cervical range of motion; OR - odds ratio;

| **Cervical Instability** | | |
| --- | --- | --- |
| **Diagnostic Test Studied** | **Studies (author last name and year)** | **Statistics Reported** |
| Magnetic Resonance Imaging (MRI) | Malhotra 2017^53^ | *Overall positive finding rate*  15.0 % (95 % CI 14.0 - 16.0 %)  *Unstable Injury Finding Rate*  0.30% (95 % CI 0.17 - 0.49 %)  *Pooled positive finding rate, weighted by the inverse of variance*  0.003%  *Obtunded Patients Positive finding rate*  0.12% (95% CI 0.03 - 0.30%)  *Pooled positive finding rate in obtunded patients weighted by inverse of variance*  0.02%  *Alert/awake patients positive finding rate:*  0.72 % (95 % CI: 0.35 - 1.32 %)  *Pooled positive finding rate in awake patients weighted by inverse of variance:*  0.01% |
|  |  |  |
|  |  |  |
|  |  |  |
|  |  |  |
|  |  |  |
|  |  |  |
